# Is Europe facing an opioid crisis like the United States? An analysis of opioid use and related adverse effects in 19 European countries between 2010 and 2018

**DOI:** 10.1192/j.eurpsy.2021.2219

**Published:** 2021-06-21

**Authors:** Mimi Pierce, Jan van Amsterdam, Gerard A. Kalkman, Arnt Schellekens, Wim van den Brink

**Affiliations:** 1 Amsterdam UMC, Location AMC, University of Amsterdam, Amsterdam, The Netherlands; 2 Department of Psychiatry, Amsterdam UMC, Location AMC, University of Amsterdam, Amsterdam, The Netherlands; 3 Department of Psychiatry, Radboud University Medical Centre, Donders Institute for Clinical Neuroscience, Nijmegen, The Netherlands; 4 Nijmegen Institute for Scientist Practitioners in Addiction, Nijmegen, The Netherlands

**Keywords:** Europe, opioid crisis, opioid mortality, prescription opioids

## Abstract

**Background:**

Given the ongoing opioid crisis in the United States (US), we investigated the opioid situation in Europe. The aims of the study are to provide an overview of trends in prescription opioid (PO) use and opioid-related adversities between 2010 and 2018 for different opioids in 19 European countries and to present a comparison with similar data from the US.

**Methods:**

A multisource database study with national data from 19 European countries evaluating trends between 2010 and 2018 in (a) PO consumption, (b) high-risk (HR) opioid users, (c) opioid-related hospital admissions, (d) opioid-related overdose deaths, (e) opioid use disorder treatment entries, and (f) patients in opioid substitution therapy (OST). Within and between-country comparisons and comparisons with data from the US were made.

**Results:**

There was considerable variation between European countries. Most countries showed increased PO consumption with the largest increase and the highest consumption in the United Kingdom (UK) compared to the rest of Europe and the US in 2018 (UK: 58,088 defined daily doses for statistical purposes/1000 population/day). In 2018, Scotland had the highest rates (per 100,000 population) of HR opioid users (16·2), opioid-related hospital admissions (118), opioid-related deaths (22·7), opioid use disorder treatment admissions (190), and OST patients (555) of all included European countries. These rates were similar or even higher than those in the US in 2018. Other countries with high rates of opioid-related adversities were Northern Ireland (synthetic and “other” opioids), Ireland (heroin and methadone), and England (all opioids). All other countries had no or little increase in opioid-related adversities.

**Conclusions:**

Apart from the British Isles and especially Scotland, there is no indication of an opioid crisis comparable to that in the US in the 19 European countries that were part of this study. More research is needed to identify drivers and develop interventions to stop the emerging opioid crisis in the UK and Ireland.

## Introduction

### The opioid crisis in the United States

The United States (US) is currently facing a serious “opioid crisis” with an increase in opioid-related deaths between 2000 and 2018 from 3 to almost 15 per 100,000 population per year [1]. The initial key driver of the crisis in the US was an increase in the use of prescription opioids (POs). Between 1999 and 2010, the sales of POs quadrupled following the inclusion of pain as the fifth vital sign, liberalization of laws governing POs, aggressive and misleading marketing by the pharmaceutical industry, and the existence of so-called “pill mills” [2,3]. Policymakers in the US responded to the opioid crisis with a national focus on reducing unnecessary prescribing, closing “pill mills,” strengthening regulatory controls and increasing treatment facilities for patients with opioid use disorders (OUD). However, opioid-related mortality continued to increase with a shift from PO-related deaths to heroin-related deaths since 2010/2011 and to illicitly manufactured fentanyl (IMF) related deaths since 2013/2014 [4–6].

### The opioid situation in Europe

The opioid situation in Europe seems to be less problematic than in the US [3,7,8]. While the number of opioid prescriptions has increased since 2009 [3], prescription rates are still much lower in most European countries than in the US, and in some countries rates are stable or declining [9]. Furthermore, according to the European Monitoring Centre for Drugs and Drug Addiction (EMCDDA), the number of opioid-related overdose deaths was stable in Europe between 2007 and 2017 at approximately 1·two opioid-related deaths per 100,000 population per year [10]. Europe is, however, heterogeneous, with some countries such as Scotland reporting a significantly higher opioid-related mortality than others [11]. Furthermore, a substantial mortality risk has been found for the nonmedical use of certain POs in some European countries [12,13]. There is also evidence that synthetic opioids are increasingly used by OUD patients seeking treatment [14].

### Relevance and aim of this paper

While there are several published studies summarizing the opioid situation in individual countries and some analyses of the European situation, an up to date summary with good quality data that provides comparisons within and between European countries and with the US is lacking [8,12]. This has several reasons. First, it is difficult to summarize already published literature due to the different parameters and methods that are used across countries. Second, relevant data are not always published in peer-reviewed papers and only exists in the “gray literature.” Finally, the Europe-wide summaries that are available, such as the annual EMCDDA reports, do not provide a detailed analysis per European country with a breakdown per opioid type [15,16]. This is unfortunate given the history of the opioid crisis in the US and we believe that data on the different types of opioids is essential for an effective monitoring of the opioid situation in Europe and for the development of targeted prevention and treatment programs. This paper thus aims to provide an overview of trends in the use of opioids and their adverse effects in Europe, using a novel approach by sourcing data from national databases in several European countries and providing separate data for the different types of opioids. Data were collected on trends between 2010 and 2018 in the following outcomes: (a) PO consumption, (b) high-risk (HR) opioid users, (c) opioid-related hospital admissions, (d) opioid-related overdose deaths, (e) opioid use disorder (OUD) treatment entries, and (f) patients in opioid substitution therapy (OST). These data were used to study trends within the European countries and to make comparisons with the US.

## Methods

### Design

Ecological study using national data from various national and international databases with information on PO use and opioid-related adversities between 2010 and 2018.

### Definitions

To collect data and interpret the findings from different countries, it is crucial to use clear definitions. Below we provide a short definition for the most relevant concepts and outcome measures.

#### Opioid crisis

Alarming situation related to opioid use, misuse, and/or opioid-related health complications, for example, increased opioid-related deaths, and treatment admissions and hospitalizations due to PO and illicit opioid use.

#### Prescription opioids

Legally manufactured opioids that can be prescribed by medical professionals mostly as analgesics or in the medical treatment of patients with an OUD. POs can also, however, be diverted and used illegally.

#### Illicit opioids

Illicitly manufactured opioids, not prescribed by medical professionals and most often used recreationally or in the context of self-medication of an OUD, including IMF and heroin.

#### Synthetic opioids

Man-made drugs (prescription and illicit) that mimic the effects of natural opioids (such as morphine), including fentanyl, fentanyl analogues, tramadol, methadone, and buprenorphine (semi-synthetic).

#### HR opioid use

Defined by the EMCDDA as “recurrent drug use that is causing actual harms to the person (including dependence, but also other health, psychological, or social problems) or is placing the person at a high probability/risk of suffering such harms (e.g., intense use of substances or use of risky combinations and/or risky routes of administration).” Estimates are mainly based on extrapolations from existing records and registers using drug use related indicators (e.g., treatment or mortality data) [17,18].

#### Defined daily dose for statistical purposes

Defined daily dose for statistical purposes. A technical measurement unit to define PO consumption. S-DDD refers to the World Health Organization (WHO) defined daily doses, that is, the assumed average maintenance dose per day for a drug used for its indication in adults [19]. The United Nations’ International Narcotics Control Board (INCB) reports opioid consumption in their annual Technical Reports in s-DDD using their own conversion factor, which is generally in line with that of the WHO [20].

### Procedures and outcomes

**Figure 1.**
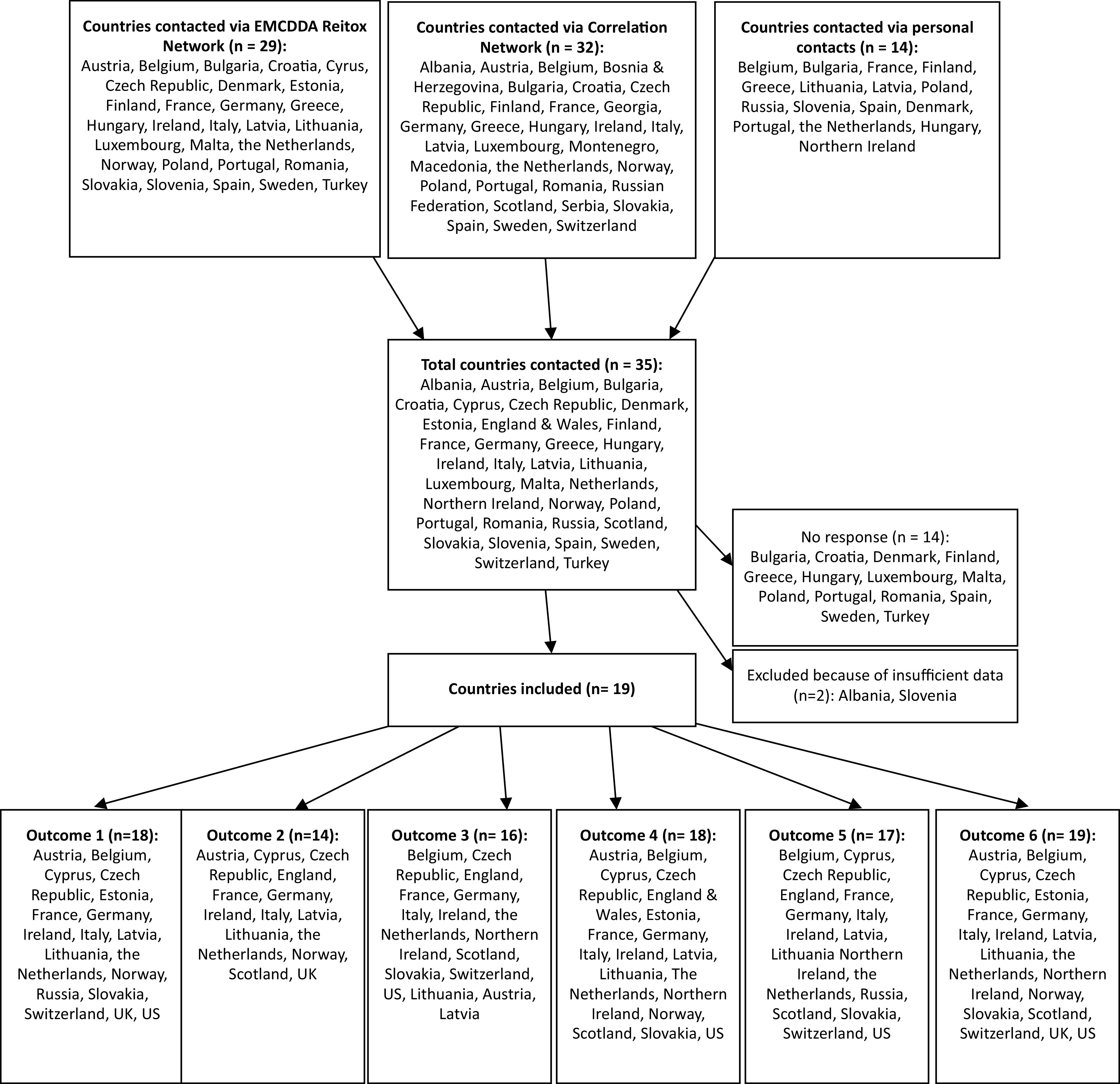
Flow chart and map of participating European countries.

A total of 36 European countries was contacted via the EMCDDA Reitox National Focal Point network, the Correlation Focal Point network, and personal contacts (Figure 1) [21,22]. Of these, 19 European countries provided sufficient data to be included in our analysis, that is, with data for at least two of the following outcomes:

#### Outcome 1: Consumption of POs

The annual Technical Reports from the INCB were used to provide data on the national consumption (in s-DDD per 1000 population per day) of different PO’s in European countries and the US [20,23–26]. See Supplement A for a detailed description of this dataset and its limitations.

#### Outcome 2: HR opioid users (most recent annual rate)

Data on the annual number of HR opioid users were taken from the most recent annual EMCDDA country reports [27]. Because the EMCDDA country reports did not cover Scotland or England, we used data from regional reports for these two areas [28–30].

#### Outcomes 3–6

Where possible data were sourced according to the ninth or tenth revision of the WHO International Classification of Diseases coding system: ICD-9 or ICD-10 (see Supplement B for relevant definitions). Rates per 100,000 inhabitants (where possible aged 15 years and older, otherwise aged 15–64 years or entire population) were calculated using population data sources per country from online statistical office databases. See Supplement C for a detailed explanation of the data.

#### Outcome 3: Opioid-related hospital admissions

Slightly different definitions were used for “all opioid-related hospital admissions” (see Supplement D). For the analysis with a breakdown per opioid type, data on opioid poisoning admissions per opioid were defined in all countries using the following ICD-10 codes: heroin *T40*·*1*, methadone *T40*·*3*, synthetic opioids (other than methadone, such as tramadol, fentanyl, and fentanyl analogues) *T40*·*4.* Poisoning admissions due to other opioids (such as codeine, morphine, hydromorphone, oxycodone, and opium) *T40*·*0*, and opium *T40*·*2*, were combined into one category (“other opioids”).

#### Outcome 4: Opioid-related overdose deaths

Data were collected on overdose deaths due to all opioids, heroin (often combined with morphine), methadone, buprenorphine, oxycodone, and other synthetic opioids (fentanyl and tramadol).

#### Outcome 5: OUD treatment admissions

Data were collected on treatment admissions (incident case entries by primary drug) due to all OUDs, and per specific OUD, including heroin, methadone, buprenorphine, oxycodone, fentanyl, and tramadol, where available. Most countries followed the EMCDDA Treatment Demand Index protocol Version 3·0 for data collection [31]. There were limitations to data coverage in many countries (see Supplement C).

#### Outcome 6: Patients in OST

Data on patients in OST (prevalent cases) were taken mainly from the annual EMCDDA country reports and otherwise from national sources in various European countries [27].

### Statistical analysis

Because the data we collected concern nationwide population data and thus do not rely on stochastic principles, it is not possible to compute variance or SE (the SE is by definition 0). We therefore did not apply statistical testing or present our findings with *p*-values or confidence intervals. The observed trends are simply whole population observations (there was no sampling of a “study population”). Moreover, the current paper focuses on robust within country changes and between-country differences, whereas relatively small variations and differences are not discussed because many of the measures that were used were not completely comparable over time or between countries. We therefore decided to use visual inspection of the data with an explicit focus on robust and policy relevant differences to identify signals of a possible opioid crisis in Europe.

## Results

The 19 European countries included in the analysis cover most European regions except for central and eastern Europe and Turkey (Figure 1). Table 1 summarizes the most recent findings for all available outcomes in these 19 European countries and the US. The text describes only the most relevant findings, that is, a brief description of the countries with the highest risks (countries with stable or low rates are thus not explicitly mentioned in the text). The level of detail in the description per country is dependent on the availability of data per country. For more details, the reader can refer to the relevant figures and Table 1.

**Table 1.** Summary of findings.

	High-risk opioid users	Consumption of all PO’s	Opioid-related hospital admissions	Opioid-related overdose mortality	Opioid treatment admissions	OST patients
	(Rate per 1000 population 15–64 years)	(Year: s-DDD per 1000,000 inhabitants per day; relative % change between 2008/2010 and 2016/2018)	(Year: rate per 100,000 population≥ 15 years)	(Year: rate per 100,000 population≥ 15 years)	(Year: rate per 100,000 population≥ 15 years)	(Year: rate per 100,000 population≥ 15 years)
Scotland		–	2010: 93*			
	16·2		2017: 118	2010: 10·9	2010: 124	2011: 589
	*(Total population)*		*(Total population)*	2018: 22·7	2018: 85	2018: 555
The United Kingdom		2008–2010: 18376				
		2016–2018: 58088				2010: 301
	8·4	216%	–	–	–	2017: 276
England		–		2010: 3·0		
	7·2		2012: 33*	2018: 4·0	2010: 119	
	*(Total population)*		2018: 35	*(England and Wales)*	2018: 84	–
Austria		2008–2010: 16315	2010: 13	2010: 4·2		2010: 279
		2016–2018: 19867	2017: 16	2018: 3·3		2018: 326
	6·3	22%	*(15–64 years)*	*(15–64 years)*	–	*(15–64 years)*
Ireland		2008–2010: 5946				
		2016–2018 7061	2010: 13	2010: 4·8	2010: 134	2010: 258
	6·2	19%	2017: 18	2017: 7·9	2018: 104	2017: 273
Italy		2008–2010: 3553				
		2016–2018: 5083	2010: 3	2010: 0·3	2013: 35	2010: 200
	6·0	43%	2017: 1	2018: 0·3	2018: 31	2018: 144
Latvia		2008–2010: 827				
		2016–2018: 2102	2014: 11	2014: 0·8	2014: 74	2010: 13
	5·7	154%	2018: 11	2018: 0·8	2018: 81	2018: 42
France		2008–2010: 8827				
		2016–2018: 8011	*2010: 8	2010: 0·5	2014: 16	2010: 304
	5·0	−9%	2018: 10	2017: 0·7	2018: 17	2017: 337
Lithuania		2008–2010: 1127				
		2016–2018: 1445	2010: 5	2010: 0·9	2013: 76	2010: 25
	3·9	28%	2018: 2	2018: 1·6	2017: 60	2018: 28
Norway		2008–2010: 9167				
		2016–2018: 11949		2010: 5·0		2010: 153
	2·7	30%	–	2018: 5·6	–	2017: 176
Cyprus		2008–2010: 704				2011: 9
		2016–2018: 1630		2010: 1·1	2011: 56	2018: 20
	2·0	132%	–	2018: 0·8	2018: 52	*(Total population)*
Germany		2008–2010: 15314	2010: 6	2010: 0·3	2010: 40	2010:95
		2016–2018: 21167	2017: 4	2016: 0·3	2018: 8	2018: 96
	2·0	38%	*(Total population)*	*(Total population)*	*(Total population)*	*(Total population)*
The Czech Republic		2008–2010: 3502				
		2016–2018: 6261	2010: 1	2010: 0·2	2010: 23	2010: 23
	1·9	79%	2018: 1	2018: 0·2	2018: 15	2018: 26
The Netherlands		2008–2010: 6565				
		2016–2018: 14621	2010: 4	2010: 0·3	2010: 10	2010: 74
	1·3	123%	2017: 10	2017: 0·9	2015: 6	2015: 37
Estonia		2008–2010: 706				
		2016–2018: 969		2010: 8·0		2010: 89
	–	37%	–	2015: 6·6	–	2018: 90
Northern Ireland		–	2010: 53*	2010: 3·6	2010: 29	2010: 45
	–		2018: 78	2017: 5·8	2016: 67	2017: 66
Switzerland		2008–2010: 11140				
		2016–2018: 16134	2012: 32*		2010: 22	2010: 227
	–	45%	2018: 23	–	2018: 31	2018: 196
Slovakia		2008–2010: 2972				
		2016–2018: 5528	2010: 7*	2010: 0·2	2014: 54	2010: 13
	–	86%	2918: 4	2018: 0·2	2018: 18	2017: 13
Russia		2008–2010: 85			2010: 61	
		2016–2018: 237			2017: 18	
	–	179%	–	–	*(Total population)*	–
Belgium		2008–2010: 22769				2010: 204
		2016–2018: 20758	2010: 6	2010: 0·3	2015: 28	2017: 183
	–	−9%	2018: 9	2016: 0·2	2018: 22	(*≥18 years)*
The United States		2008–2010: 18376	2010: 197*	2010: 6·8	2010: 143	2011:393
		2016–2018: 17853	2017: 300	2018: 14·6	2017: 210	2017: 417
	–	−6%	*(Total population)*	*(Standard population)*	*(Total population)*	*(Total population)*

*Note:* –, no data available. Opioid-related hospital admissions: *same definition used to define data. If a different population sample (not 15 years and older) was used to calculate the rates this is given in brackets following the data from that country.

Abbreviations: OST, opioid substitution therapy/treatment; PO, prescription opioids; S-DDD, defined daily dose for statistical purposes.

### Outcome 1: Trends in PO consumption 2010–2018

**Figure 2.**
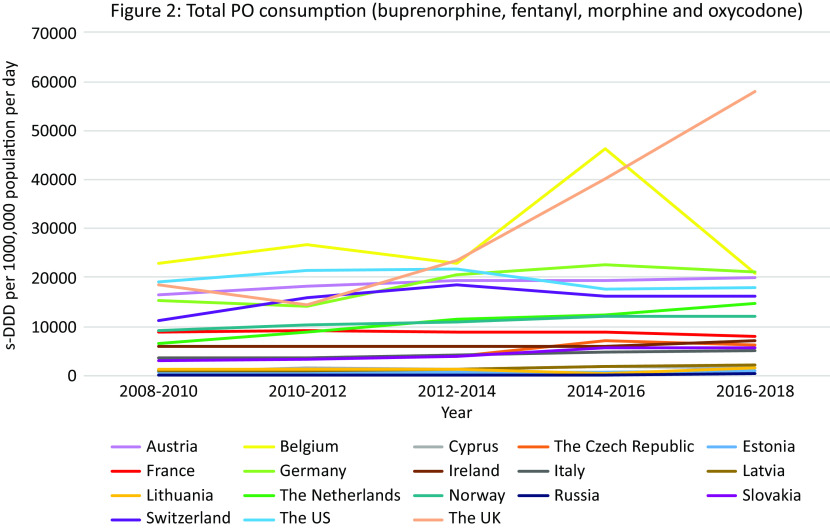
PO consumption. Different scales are used on the *Y*-axis. In the case of missing data, the available data points were connected with a line. The data presented in tables and figures as 2008–2010 are from the 2011 INCB report, the data presented as 2010–2012 from the 2013 INCB report, 2012–2014 from 2015 INCB report, 2014–2016 from the 2017 INCB report, and 2016–2018 from the 2019 INCB report.

PO consumption data from the INCB was available for 17 European countries and the US (Figure 2).

#### Overall PO consumption

Apart from Belgium and France, all European countries demonstrated increasing trends in PO consumption over the observation period (Figure 2). While the initial rate in the UK was lower than in the US, the largest increasing trend in opioid consumption was seen in the UK (216% in the last decade) resulting in the highest consumption of POs compared to the rest of Europe and the US in 2018 (58,088 s-DDD per 1000,000 population per day). Belgium also had a high initially stable rate followed by a peak in 2014–2016 (46,296 s-DDD per 1000,000 population per day) and then a decrease in 2016–2018. Other European countries with consumption rates higher than the US were Germany (21,667 s-DDD per 1000,000 population per day in 2016/2018) and Austria (19,876 s-DDD per 1000,000 population per day in 2016/2018).

#### Opioid-specific consumption

An increasing trend in *buprenorphine* consumption was observed in Austria, Cyprus, the Czech Republic, Estonia, Germany, Ireland, Latvia, the Netherlands, Norway, Slovakia, Switzerland, the US, and the UK (Supplement Figure S2b–e).

An increasing trend in *fentanyl* consumption was observed in Austria, Belgium, Cyprus, the Czech Republic, Estonia, Germany, Italy, Latvia, Lithuania, the Netherlands, Norway, Russia, Slovakia, and the UK.

An increasing trend in *morphine* consumption was seen in Austria, Estonia, Ireland, the Netherlands, Russia, and Switzerland.

An increasing trend in *oxycodone* consumption was seen in Austria, Belgium, Cyprus, the Czech Republic, Estonia, France, Germany, Ireland, Italy, Lithuania, the Netherlands, Norway, Russia, Slovakia, and Switzerland.

#### Summary

The highest consumption of POs compared to the rest of Europe and the US in 2018 was in the UK, where specifically an increasing trend in buprenorphine and fentanyl consumption was seen between 2008/2010 and 2016/2018.

### Outcome 2: HR opioid users

A recent annual rate of HR opioid users was available for 14 European countries (Table 1). The highest rate was seen in Scotland (16·two per 1000 population 15–64 years old), followed by England (7·two per 1000 population 15–64 years old), and other European countries (1.3–6.3 per 1000 population 15–64 years old).

### Outcome 3: Trends in opioid-related hospital admissions 2010–2018

#### Total opioid-related hospital admissions in Europe and the US

**Figure 3.**
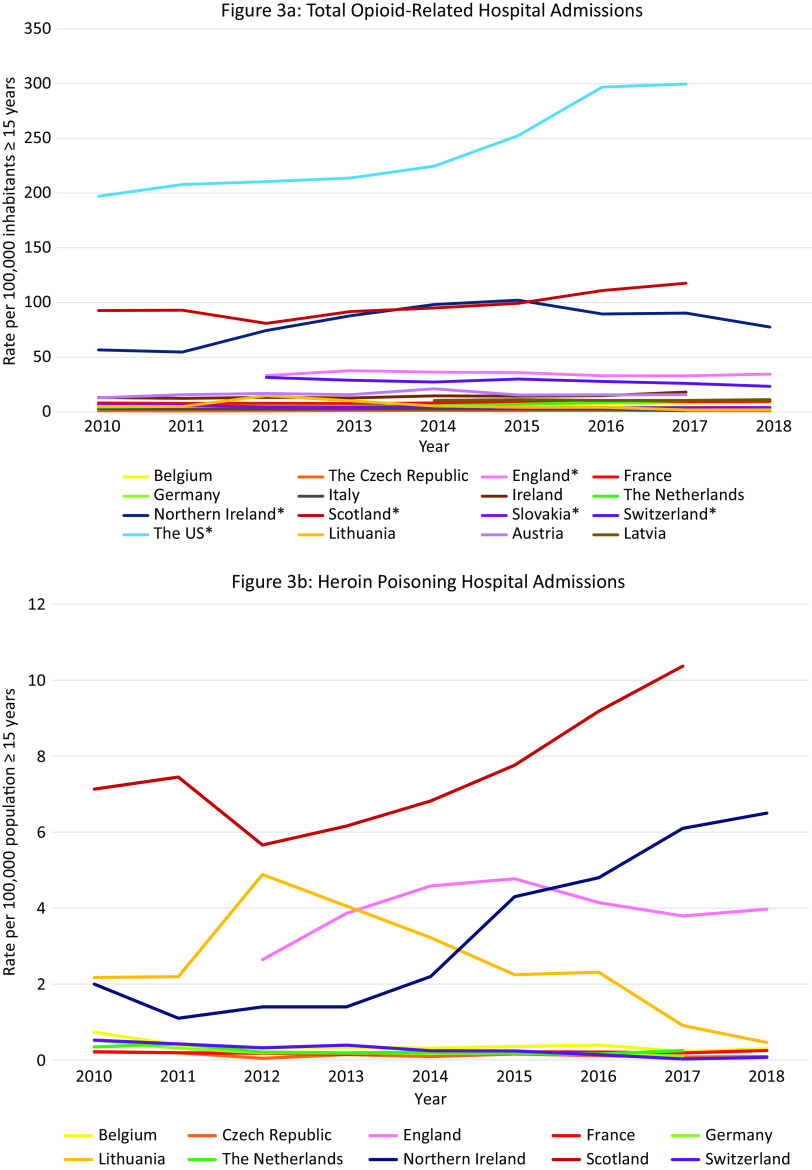
Opioid-related hospital admissions*.* Different scales are used on the *Y*-axis. In the case of missing data, the available data points were connected with a line. The *Y*-axis refers to population age, for example, inhabitants aged ≥15 years. *Similar definition of data, all other countries different definitions (see Supplement D).

Data were available from 16 countries, however different methods and definitions limit Europe-wide comparisons (see Supplement D) (Figure 3A). The US had the highest rates of opioid-related hospital admissions between 2010 and 2017 (197 per 100,000 population in 2010, 300 in 2018). Within Europe, Scotland and Northern Ireland had the highest total opioid-related hospital admission rates and increasing trends over the observation period, however the rates still remained lower than in the US (Scotland: 93 per 100,000 population in 2010, 118 in 2017; Northern Ireland: 57 per 100,000 population in 2010, 78 in 2018). Regarding trends in other European countries, opioid-related hospital admission rates in the Czech Republic, England, and Latvia remained stable. A decreasing trend was seen in Italy, Germany, Slovakia and Switzerland, and an increasing trend in Austria, Belgium, France, Ireland, and the Netherlands. While in Lithuania a decreasing trend was observed, there was a sharp increase between 2011 and 2012 from 5 per 100,000 population to 15 per 100,000 population, followed by a decrease again to 5 in 2014.

**Figure:**
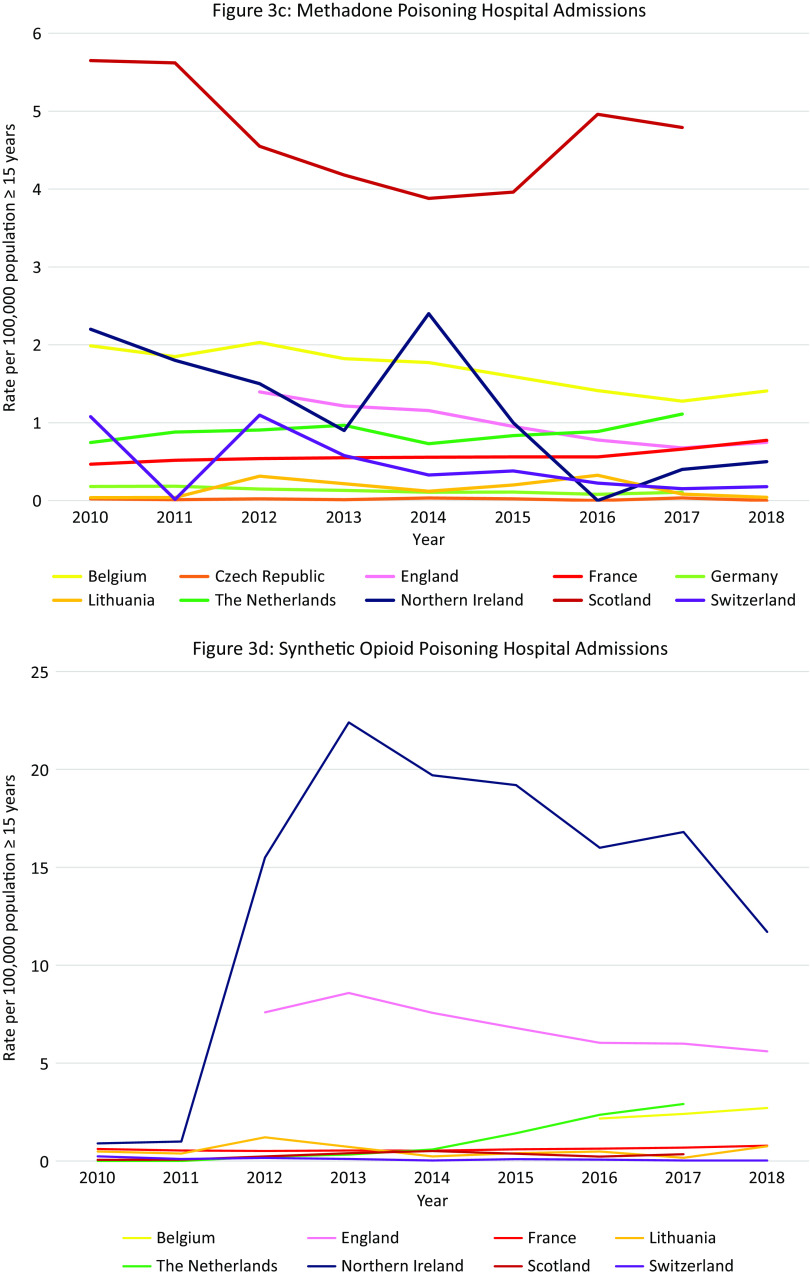

**Figure:**
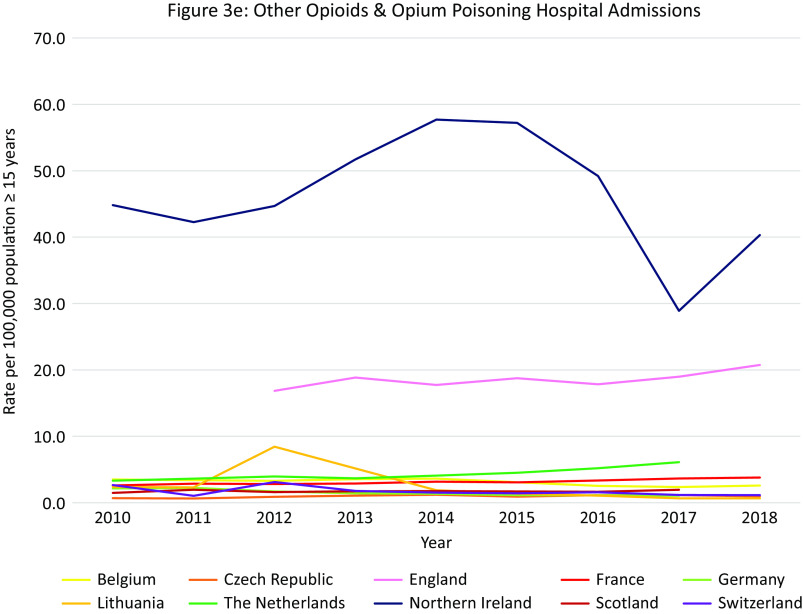

#### Opioid-related hospital admissions per opioid in Europe

Data were available from 10 European countries (Figure 3B–E). The highest rate of *heroin* poisoning admissions was in Scotland, with an increasing trend since 2012 (7·1 per 100,000 population in 2010 vs. 10·4 in 2017). An increasing trend was also observed in Northern Ireland (2·0 per 100,000 population in 2010 vs. 6·5 in 2018), and a smaller increase in England (2·6 per 100,000 population in 2012 vs. 4·0 in 2018). A high rate was also seen in Lithuania in 2012 (4·9 per 100,000 population) followed by a decrease (0·5 per 100,000 population in 2018).

The highest *methadone* poisoning hospital admission rate was in Scotland (5·7 per 100,000 population in 2010 and 4·8 in 2017). In Northern Ireland, a peak was observed in 2014 followed by a decrease (2·4 per 100,000 population in 2014 vs. 0·5 in 2018). A decreasing trend was seen in Belgium (2·0 per 100,000 population in 2010 vs. 1·4 in 2018) and England (1·4 per 100,000 population 2012 vs. 0·7 in 2018). An increasing trend was seen in the Netherlands (0·7 per 100,000 population in 2010 vs. 1·1 in 2017), and France (0·5 per 100,000 in 2010 vs. 0·8 in 2018). Following peaks in 2010 and 2012 (1·1 per 100,000 population), a decreasing trend was seen in Switzerland.

The highest *synthetic opioid* poisoning admission rate was in Northern Ireland, with a sharp increase between 2011 and 2013 from 1·0 to 15·5 per 100,000 population, followed by a gradual decrease (11.7 per 100,000 population in 2018). High rates were also observed in England, but with a decreasing trend (7·6 per 100,000 population in 2012 vs. 5·6 in 2018). An increasing trend was observed in the Netherlands between 2011 and 2017 (0·0 vs. 2·9 per 100,000 population), and in Belgium (2·2 per 100,000 in 2016 vs. 2·7 in 2018).

The highest “*other opioid*” poisoning hospital admission rate was in Northern Ireland (44·8 per 100,000 population in 2010, 40·3 in 2018). High rates were also seen in England (16·8 per 100,000 population in 2012, 20·7 in 2018). An increasing trend was observed in the Netherlands (3·3 per 100,000 population in 2010, 6·1 in 2018).

#### Summary

The highest rates of opioid-related hospital admissions were seen in the US, followed by Scotland and Northern Ireland where much higher rates were observed than in other European countries. In Scotland poisonings mainly involved heroin and methadone, whereas in Northern Ireland this was “other opioids,” synthetic opioids and—increasingly–heroin. High rates of “other opioids,” synthetic opioids and heroin poisonings were also seen in England.

### Outcome 4: Trends in opioid-related overdose deaths 2010–2018

#### Total opioid-related overdose deaths in Europe and the US

**Figure 4.**
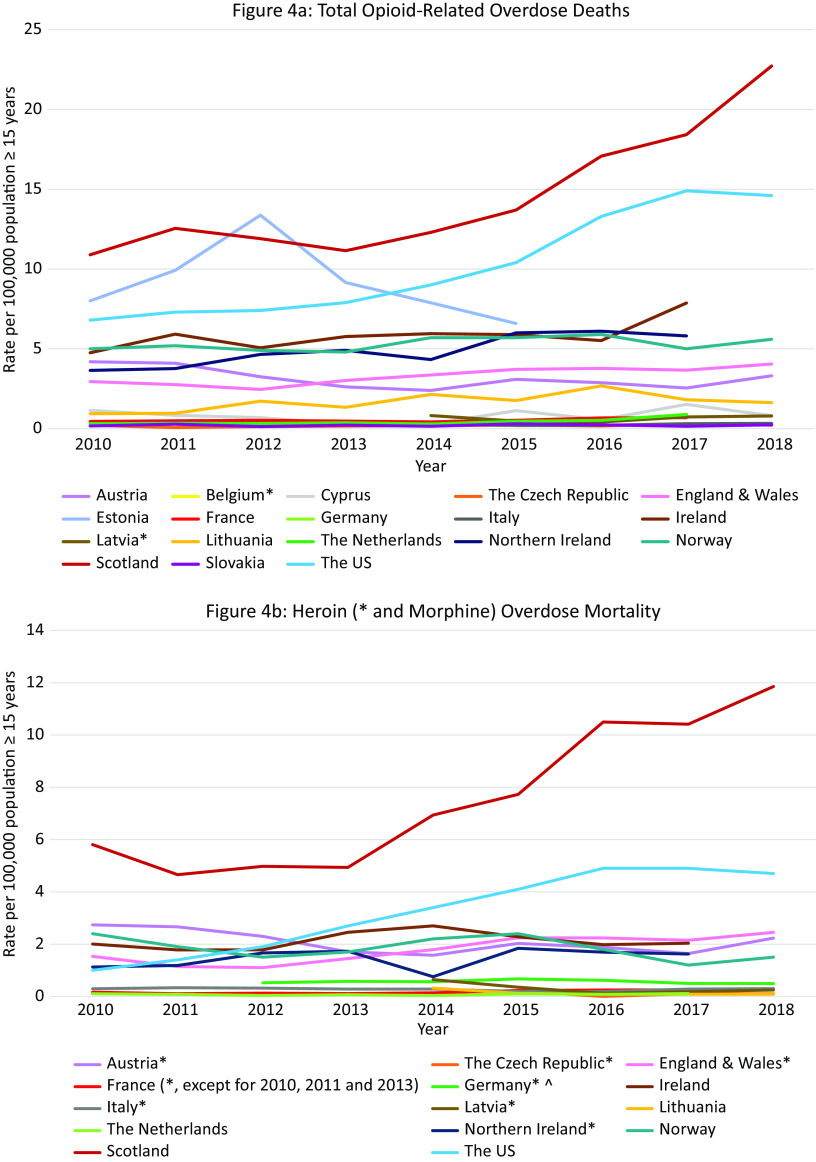
Opioid-related overdose deaths. Different scales are used on the *Y*-axis. In the case of missing data, the available data points were connected with a line. The *Y*-axis refers to population age, for example, inhabitants aged ≥15 years. *There were a number of underreporting issues (see Supplement C).

Data were available from 18 countries (Figure 4A). Within Europe the highest mortality rates were in Scotland (10·9 per 100,000 in 2010 and 22·7 in 2018), where opioid-related mortality increased by 108·6% and exceeded the opioid-related mortality in the US in 2018. High rates were also observed in Estonia with a peak in 2012 of 13·4 per 100,000 population followed by a decreasing trend (6·6 in 2015), Ireland with an increasing trend between 2010 and 2017 peaking at 7·9 per 100,000 population in 2017, Northern Ireland with an increasing trend between 2010 and 2016 (3·6 per 100,000 population vs. 6·1) followed by a slight decrease in 2017 (5·8), and Norway with a peak of 5·9 per 100,000 population in 2016 followed by a decreasing trend (5·6 in 2018).

**Figure:**
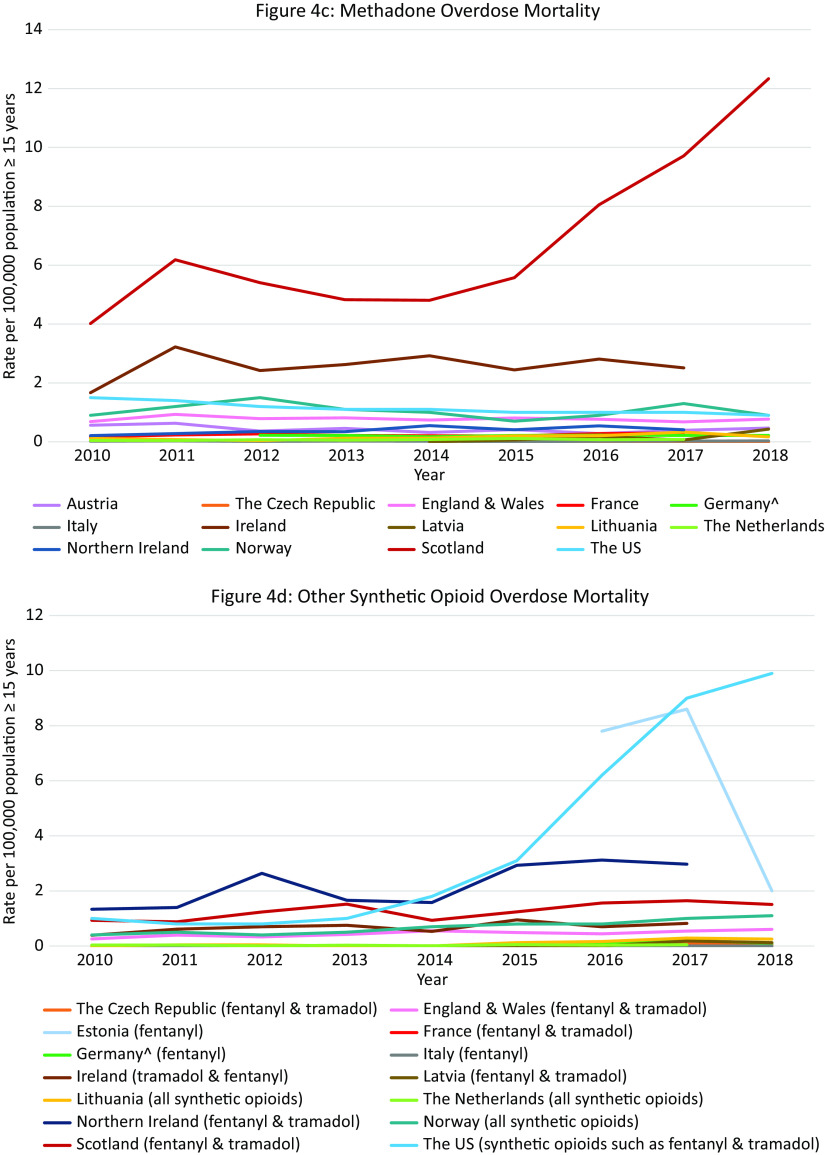

**Figure:**
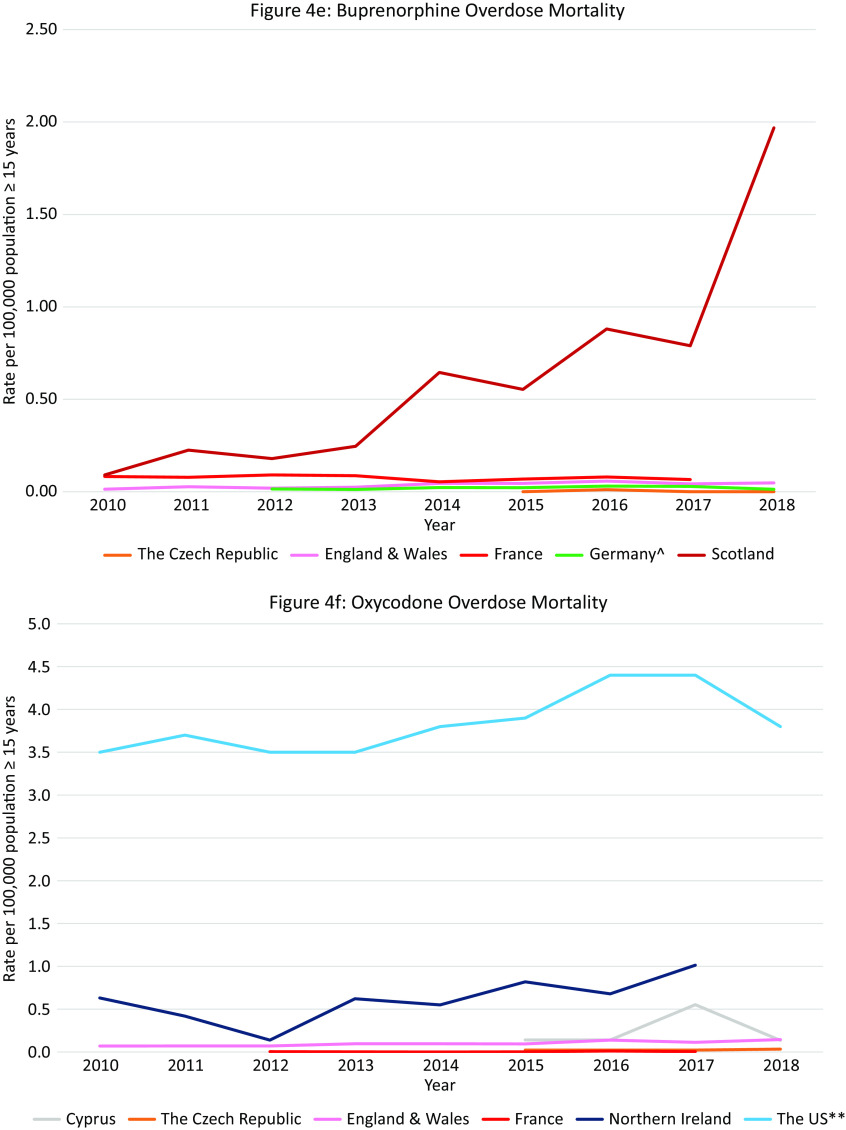

#### Overdose mortality per specific opioid in Europe and the US

Data were available from 14 countries (Figure 4B–F). The highest *heroin* (and morphine) mortality rates were in Scotland where an 104% increase was seen over the 8-year period (5·8 per 100,000 population in 2010 vs. 11·9 in 2018), exceeding rates in the US in the same period (1·0 per 100,000 population in 2010, 4·7 in 2018).

The highest *methadone* mortality rates were also in Scotland where a 207% increase was observed over the 9-year period (4·0 per 100,000 population in 2010 vs. 12·3 in 2018), again exceeding the rates seen in the US (1·5 per 100,000 population in 2010, 0·9 in 2018). Rates in Ireland also exceeded those in the US (1·7 per 100,000 population in 2010, 2·5 in 2017).

The highest rate of *synthetic opioid* mortality within Europe was in Estonia (fentanyl) where data were only available between 2016 and 2018 with a peak at 8·6 per 100,000 population in 2017, followed by a steep decline in 2018 to 2·0 per 100,000 population. Thus, the peak rate came close to the rates seen in the US in this period (9·0 and 9·9 per 100,000 population in 2017 and 2018 respectively, fentanyl and tramadol grouped together). High rates and an increasing trend were also observed in Northern Ireland (1·3 per 100,000 population in 2010 vs. 3·0 in 2018, fentanyl and tramadol grouped together).

The highest rate of *buprenorphine* mortality within Europe was in Scotland with a steep increase between 2010 and 2018 (0·1 per 100,000 population in 2010 vs. 2·0 in 2018).

The highest rate of *oxycodone* mortality within Europe was in Northern Ireland with an increasing trend reaching 1·0 per 100,000 population in 2018. The rate did not reach the rate in the US in the same period (3·8 per 100,000 population in 2018), however the US data also included morphine and hydrocodone.

#### Summary

Scotland was a clear outlier, with more opioid-related overdose deaths than in the US during the entire observation period. However, unlike in the US where synthetic opioids are currently the leading cause of opioid-related deaths, a breakdown per opioid type revealed that heroin and methadone were the main drugs involved in opioid-related mortality in Scotland. Apart from in Northern Ireland, synthetic opioids did not appear to be currently problematic in terms of mortality in Europe, despite a transient increase in Estonia. Ireland warrants attention due to relatively high rates of methadone overdose deaths.

### Outcome 5: Trends in OUD treatment admissions 2010–2018

#### Total opioid treatment admissions in Europe and the US

**Figure 5.**
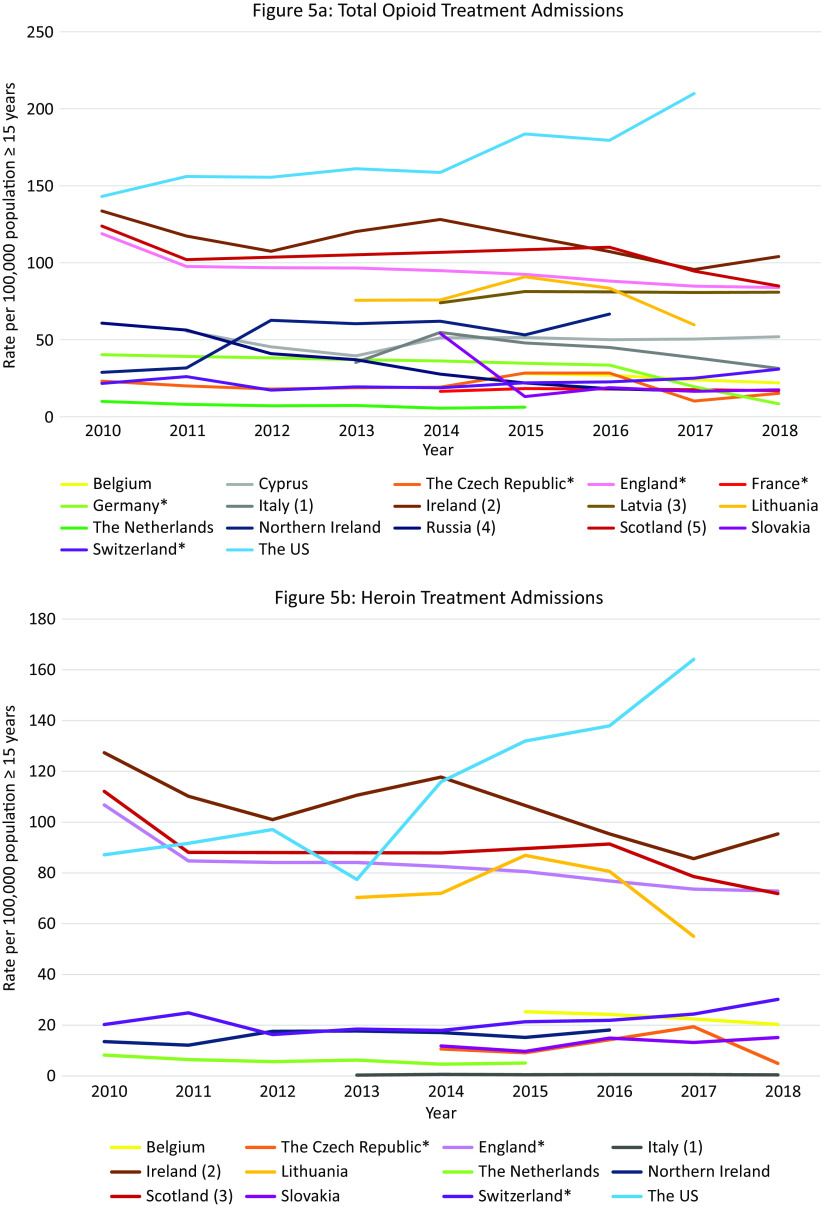
Opioid treatment admissions. Different scales are used on the *Y*-axis. The *Y*-axis refers to population age, for example, inhabitants aged ≥15 years. In the case of missing data, the available data points were connected with a line. *Limitations to data coverage resulting in underestimation (see Supplement C for more detailed explanation). (a) Only outpatient treatments recorded. (b) Overestimate: patients could be counted twice in the database if they received treatment at the same center more than once per calendar year. (c) Overestimate: all patients in treatment (not treatment admissions). (d) All patients hospitalized in psychiatric hospitals for opioid addiction. (e) Overestimate: all assessments for treatment.

Data were available from 17 countries (Figure 5A). Within Europe, Ireland, and Scotland had the highest opioid treatment admission rates with decreasing trends over the observation period (Ireland: 134 per 100,000 population in 2010, 104 per 100,000 population in 2018; Scotland: 124 per 100,000 population in 2010 and 85 in 2018). However, these rates did not reach the extremely high and increasing treatment rates in the US (143 per 100,000 population in 2010 to 210 per 100,000 population in 2017).

**Figure:**
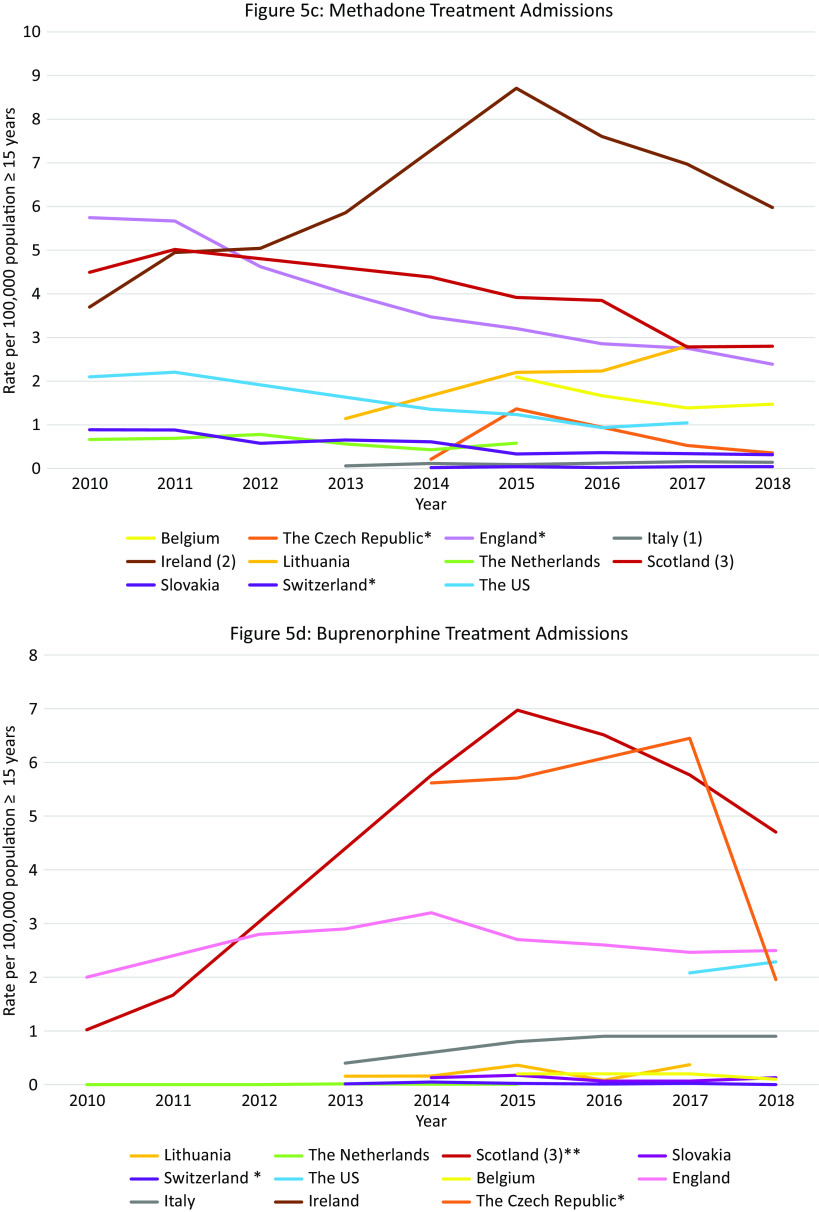

**Figure:**
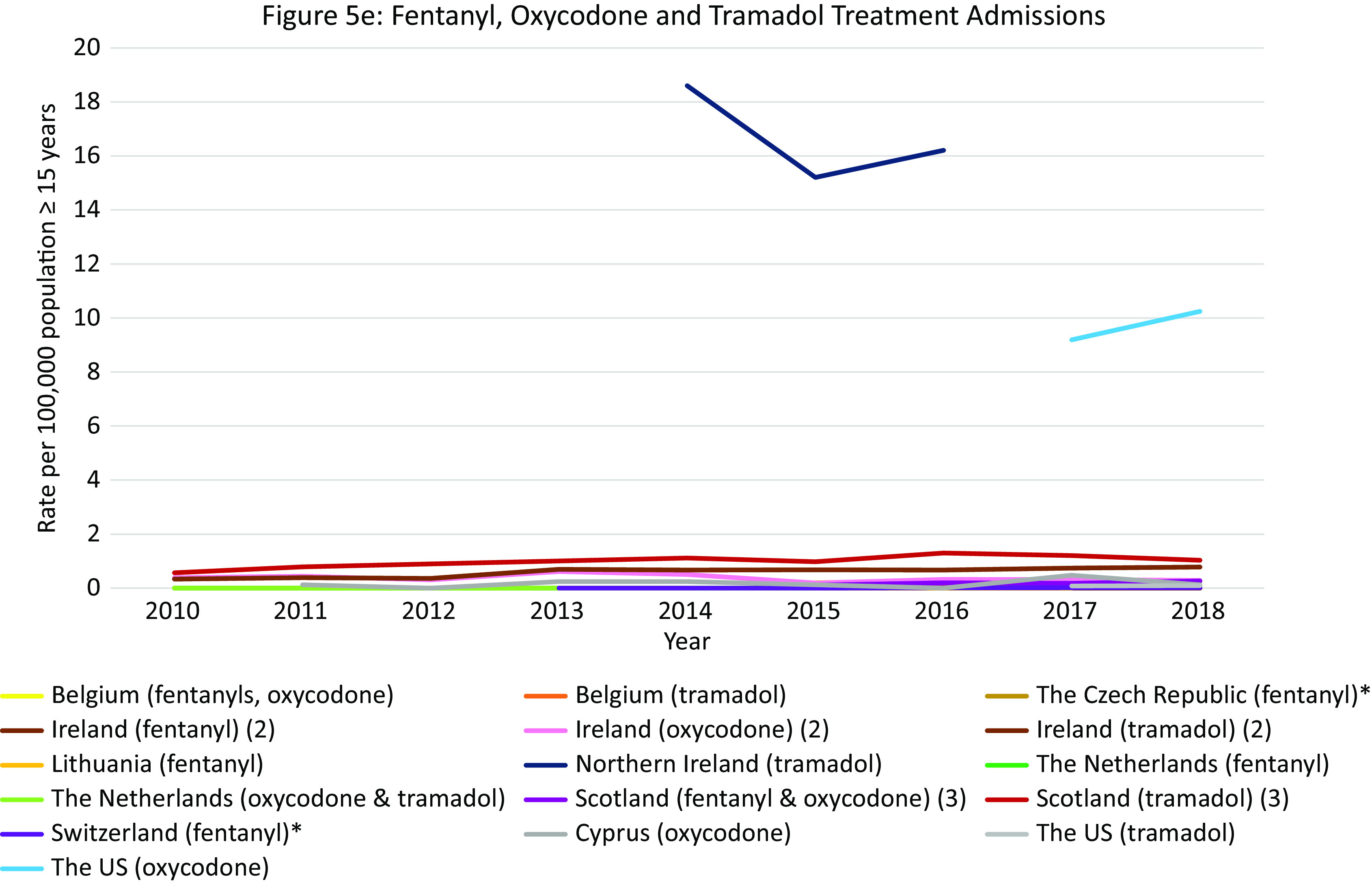

#### Opioid treatment admissions per opioid in Europe and the US

Data were available from 12 countries (Figure 5B–E). Ireland had the highest *heroin* admission rates which decreased from 127·4 per 100,000 population in 2010 to 101 in 2012, peaked again at 117·8 in 2014, and then decreased again to 95·3 in 2018. Scotland and England also had high heroin treatment admission rates, which were rather stable over time (between 80 and 110 admissions per 100,000 population). The admission rates in the US were initially lower than in the UK (2010: 87·2 per 100,000), but started to increase from 2014 and reached an all-time high in 2018 (137·9 per 100,000), which was substantially higher than the rates in Ireland (99 per 100,000 population), Scotland (91·4 per 100,000), and England (72·9 per 100,000 population) at that time.

Scotland, England, and Ireland initially demonstrated very high *methadone* admission rates (2010: England 5·7, Scotland 4·5, Ireland 3·7 per 100,000 population), exceeding those in the US (2·1 per 100,000 population in 2010). However, following a steep decrease during the observation period, rates in England and Scotland were similar to those in the rest of Europe and the US (Scotland 2018: 2·8; England 2018: 2·4; US 2017: 1·0 per 100,000 population). Rates in Ireland, however, increased during the observation period, peaking in 2015 at 8·7 per 100,000 population and then decreasing slightly to 6·0 per 100,000 population in 2018. Other countries with rates exceeding those in the US were Belgium (decreasing trend from 2·1 per 100,000 population in 2015 to 1·5 in 2018), Lithuania (increasing trend from 1·1 per 100,000 population in 2013 to 2·8 in 2017), and the Czech Republic (short peak in 2015 of 1·4 per 100 000 population).

Between 2010 and 2015 there was a steep increase in *buprenorphine* admissions in Scotland (7·0 per 100,000 population in 2015) followed by a gradual decline, but rates remained higher than those observed in the US (Scotland 4·7 per 100 000 population in 2018, US 2·3 in 2018). High buprenorphine admission rates exceeding those in the US were also observed in the Czech Republic between 2014 and 2017 (5·6 per 100,000 population and 6·4, respectively) followed by a steep decline in 2018 (2·0 per 100 000 population), and in England (2·0 per 100,000 population in 2010 vs. 2·6 2016).

Except for Northern Ireland, rates of “*fentanyl, oxycodone or tramadol*” admissions were rather low in Europe. Regarding tramadol, high treatment admissions rates were observed in Northern Ireland between 2014 and 2017 (18·6–16·2 per 100,000 population). In Scotland rates remained approximately 1·0 per 100,000 population, and in Ireland rates increased from 0·3 per 100,000 population in 2010 to 0·8 in 2018. These were, however, higher than the tramadol treatment rates observed in the US in 2017 and 2018 (0·1 per 100,000 population). Regarding fentanyl and oxycodone, in Scotland, the treatment admissions increased from 0·1 per 100,000 population in 2015 to 0·3 in 2018. Importantly, the oxycodone admission rates did not reach the extremely high rates seen in the US in 2017 and 2018 (9·2 and 10·2 per 100,000 population, respectively).

#### Summary

Again, within Europe Scotland was an outlier with extremely high opioid treatment admission rates, exceeding those in the US up until 2017. While the overall rate was relatively stable, shifts occurred in the contributing drugs. While heroin remained the largest contributor, it was initially followed by methadone but ultimately by buprenorphine in 2018. Other European countries that warrant attention include Ireland and England (very high heroin rates, relatively high methadone rates), Northern Ireland (tramadol) and the Czech Republic (buprenorphine).

### Outcome 6: Trends in OST in Europe and the US 2010–2018

**Figure 6.**
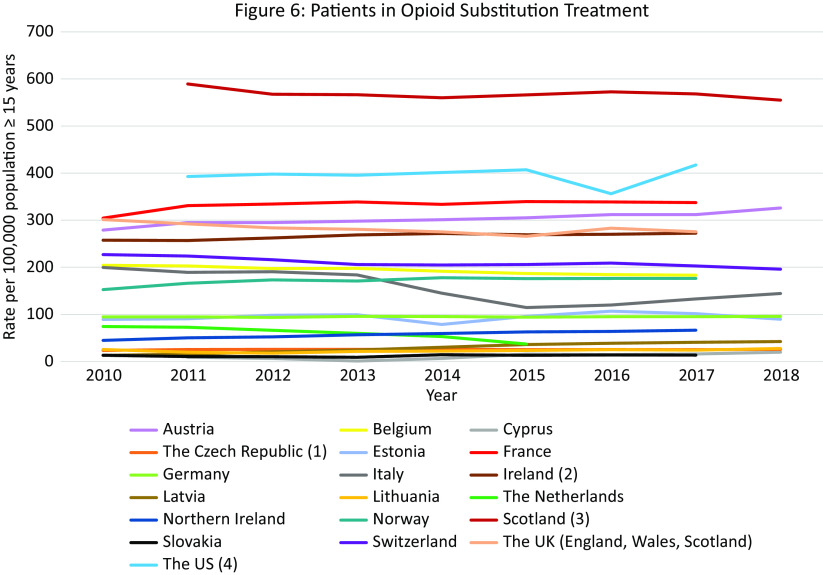
Patients in OST. Different scales are used on the *Y*-axis. The *Y*-axis refers to population age, for example, inhabitants aged ≥15 years. In the case of missing data, the available data points were connected with a line. (a) Underestimation due to nonregistration of all patients receiving OST. (b) Data excludes small number of patients receiving OST with suboxone. (c) An estimate based upon Community Health Index numbers captured on prescriptions. (d) Underestimate because patients prescribed buprenorphine through an independent DATA 2000-waivered medical practitioner or naltrexone through an independent practitioner not affiliated with a substance abuse treatment facility are not represented in the dataset.

Data were available from 19 countries. The rate in most countries remained relatively stable over the observation period. The highest OST rates between 2011 and 2018 were in Scotland (555 per 100,000 population in 2018) followed at a distance by the US, France, Austria, the UK as a whole, and Ireland (Figure 6). Scotland was the only country with rates higher than those seen in the US in the same period.

## Discussion

The current analysis provides evidence for an increasing trend in PO consumption in almost all included European countries, with the largest increase in the UK. Regarding opioid-related adverse effects there was considerable variation between European countries. While most countries demonstrated low stable rates of opioid-related harms, we did identify increases in opioid-related harms in some European countries. With the exception of the British Isles and especially Scotland, however, these increases were not comparable to those in (most states of) the US in the past decade. This is in line with our assessment 5 years ago, when we concluded that the risk of an opioid overdose crisis in Europe was small [5].

There are several differences between the US and Europe that may have prevented the development of an opioid crisis in European countries. First, marketing of oxycodone directly to patients is often cited as one of the reasons for the development of increased prescribing and thus increased opioid-related harms in the US, whereas marketing of drugs directly to patients is prohibited in Europe [32,33]. Second, while IMF is a serious/dominant problem in the US, our data suggest that except for Estonia, IMF is not a problem in Europe [34]. IMF was an important driver of the third phase of the opioid crisis in the US, because (car)fentanyl is a very potent opioid with a high abuse liability and because it is regularly used as an adulterant of heroin and cocaine, or sold as a falsified PO, resulting in fatal incidents in drug users unaware of the presence of a highly potent opioid [6]. Third, there are important differences between the US and Europe in the availability of “street” PO’s (high diversion and doctor shopping in the US vs. Europe) [35]. Finally, the availability of free-of-charge OUD treatments, including OST, is much lower in the US compared to most European countries [36–38].

Our current analysis revealed large variations in the opioid situation within Europe. Most importantly, we observed high PO consumption in the UK and high and often increasing rates of opioid-related adverse effects in the British Isles (Scotland, Northern Ireland, Ireland, and England). Considering that these countries share many cultural, socioeconomic, and health care characteristics, our data suggest a fundamental sociocultural and/or policy problem in this region. Research and action are warranted to prevent further escalation of a serious regional opioid crisis on the British Isles.

Interestingly, we also observed regional differences within the British Isles. Heroin and methadone were mainly responsible for opioid-related harms in Scotland and Ireland. In Northern Ireland mainly synthetic opioids other than methadone (mainly tramadol) and “other opioids” were most important. In England, a mix of different opioids were responsible for different opioid-related harms. These findings call for a multifactorial in-depth analysis of regional differences in the drivers of the British opioid situation, including differences in market dynamics related to the availability, purity, and price of different opioids.

Scotland was consistently an outlier in all our analyses, with the highest rate of HR opioid users, the highest total opioid-related hospital admission rates, the second highest opioid treatment admission rates, the largest number of OST patients, and an extremely high rate of opioid-related overdose deaths, even exceeding the rate seen in the US. Our findings are in line with the recent literature suggesting that Scotland currently faces a true opioid crisis, comparable in magnitude to that in the US [10,39]. The history, the types of opioids, and the mechanisms involved in the opioid crisis in these two countries are, however, different. In a recent study, we concluded that specific drivers for the opioid crisis in Scotland seem to include frequent polydrug use (opioids combined with benzodiazepines or gabapentinoids), steeper aging of the drugs using population in the past two decades, and low treatment coverage and efficiency in Scotland compared to England/ Wales [40]. Based on these findings, several recommendations can be made to further prevent opioid-related problems in Europe, including evidence-based guidelines for pain treatment, evidence-based improvements to establish an efficient addiction treatment system, and raising awareness about the dangers of combining opioids with other central depressants [41,42].

Our analysis also revealed some transient increases in opioid-related harms that reflect the complexity of Europe’s contemporary drug market, heterogeneity in national regulations and highlight the importance of early detection and monitoring of opioid supply chains and availability. In the Czech Republic high buprenorphine treatment admission rates were observed in 2014, 2015, and 2017 [43]. This local epidemic is attributed to reduced availability of and waiting lists for buprenorphine OST since 2003, local changes in the drug scene resulting in increased demand and higher prices of illegal heroin, and increased availability of illegal and diverted buprenorphine [43–45]. A second example is Estonia, where we observed high rates of fentanyl-related mortality peaking in 2017 (nearly reaching the rates observed in the US), in line with the already observed fentanyl epidemic in Estonia. Fentanyl became the most commonly used opioid among people who inject drugs in Estonia between 2000 and 2010, following a severe disruption in heroin availability due to stricter controls on the Estonian–Russian border [15,46].

### Strengths and limitations

While there have been several articles and reports published about opioid-related deaths and hospital admission in specific countries in Europe due to specific opioids, there have been no systematic data analyses by country or opioid type [47,48]. This is one of the major strengths of the current study. We used the novel approach of creating a large database using data from national sources in several European countries on a broad range of opioid-related topics with a breakdown per opioid type. In general, however, comparisons across and within countries were limited by variations in national methods used and a lack of data from half of all European countries. Furthermore, our data provided no information about the source of the opioid involved or the nature of the opioid-related adversity (e.g., mistaken use of prescription or illicit opioid vs. suicide attempt).

Regarding our measure of PO consumption, questions remain about the validity of the INCB data (see Supplement A). Furthermore, although there was a parallel increase in PO consumption and opioid-related adversities in the UK, this pattern was absent in other countries, and therefore parallel patterns cannot automatically be interpreted as causal. We also like to note that increased PO prescribing is not necessarily problematic and may instead be a reflection of improved pain management. Similarly, an increase in buprenorphine prescription may indicate improved treatment access for OUD.

Importantly, our between-country comparisons of opioid-related hospital admission rates were limited by variation in definitions. Furthermore, only nonfatal overdose hospital admissions (primary diagnosis) were included, whereas overdoses treated by other facilities (e.g., poison information centers) were not covered. Finally, the growing implementation of Take-Home Naloxone kits (THN) may have decreased the presentation of opioid overdoses to hospitals, leading to an underestimation [49].

Our mortality data were difficult to interpret because some countries have more resources available to conduct toxicological analyses than others. Even the trends within countries may be problematic because new technical developments in forensic and toxicological analyses influence the findings. This is particularly important for the detection of new emerging highly potent opioids such as fentanyl and its analogues [50].

While our OST data could be interpreted as a reflection of the number of opioid dependent patients, it is known that large variations exist in accessibility and availability and thus treatment coverage in Europe [51,52]. Finally, while the highest rates of opioid-related harms were seen in Scotland, Scotland also had the highest rate of HR opioid users. Future research should adjust for this potential confounding factor and thus help clarify whether the opioid crisis in Scotland is driven by factors causing a higher prevalence of HR opioid users, factors causing more adverse effects in each HR opioid user or both.

## Conclusions and Recommendations

Apart from the British Isles, and especially Scotland, there appears to be no evidence for an opioid crisis in the European countries studied that is similar in magnitude or nature to the crisis in the US. The long-lasting and ongoing crisis in Britain is likely to be related to regional socioeconomic and cultural demand issues and problems in the treatment system. Transient increases in specific opioid-related problems observed in the Czech Republic and Estonia are on the other hand more likely to be related to temporary opioid supply issues, due to changes in the illegal market or a lack of treatment availability. These trends also reveal the potential risk of excessive restrictions of POs, whereby a limit in the supply may lead to a paradoxical rise in substitute use of illicit opioids. Considering the diversity seen across Europe, our findings call for national and regional actions rather than Europe-wide strategies to monitor and address opioid-related problems in the future.

## Data Availability

Data are available from the authors at mimi.j.pierce@gmail.com.

## Abbreviations

EMCDDA: European Monitoring Centre on Drugs and Drug Abuse
HAT: heroin assisted treatment
HR: high-risk
ICD-10: International Classification of Diseases coding system
IMF: illicitly manufactured fentanyl
INCB: International Narcotics Control Board
OST: opioid substitution therapy/treatment
OUD: opioid use disorder
POs: prescription opioids
s-DDD: defined daily dose for statistical purposes
THN: take-home naloxone kits
US: United States
UK: United Kingdom
WHO: World Health Organization

## Supplementary Materials

To view supplementary material for this article, please visit http://dx.doi.org/10.1192/j.eurpsy.2021.2219. click here to view supplementary material
