# Supplementary material for: Is Europe facing an opioid crisis like the United States? An analysis of opioid use and related adverse effects in 19 European countries between 2010 and 2018
Source: Eur Psychiatry. 2021 Jun 21;64(1):e47. doi: 10.1192/j.eurpsy.2021.2219 (PMC8316471; doi:10.1192/j.eurpsy.2021.2219)
Supplement: Supplementary file 1 [file epasup.zip › S0924933821022197sup002.docx]

**Supplement Figures and Tables**

*Figures 2a-2e: PO Consumption*

Please note: different scales are used on the Y-axis. In the case of missing data, the available data points were connected with a line.

The data presented in tables and figures as 2008-2010 is from the 2011 INCB report, the data presented as 2010-2012 from the 2013 INCB report, 2012-2014 from 2015 INCB report, 2014-2016 from the 2017 INCB report and 2016-2018 from the 2019 INCB report.

*Table 7 INCB vs local database data, s-DDD per 1000,000 inhabitants per day*

|  | **2014** | | **2015** | | **2016** | | **2017** | | **2018** | |  |  |  |
| --- | --- | --- | --- | --- | --- | --- | --- | --- | --- | --- | --- | --- | --- |
|  | **ND** | **INCB** | **ND** | **INCB** | **ND** | **INCB** | **ND** | **INCB** | **ND** | **INCB** | **Trend 2014-2018 ND** | **Trend 2014- 2018 INCB** | **Considerable difference in data ND vs INCB** |
| **Morphine** |  |  |  |  |  |  |  |  |  |  |  |  |  |
| *Netherlands GIP databank* | 370 | 309 | 408 | 338 | 419 | 293 | 430 | 299 | 446 | 269 | Increase | Decrease |  |
| *Norwegian Prescription Database data* | 752 | 665 | 705 | 642 | 709 | 634 | 723 |  | 637 |  | Decrease | Decrease |  |
| *Norwegian Wholesale data* | 1170 | 665 | 1050 | 642 | 1110 | 634 | 1080 |  | 1060 |  | Decrease | Decrease |  |
| **Oxycodone** |  |  |  |  |  |  |  |  |  |  |  |  |  |
| *Netherlands GIP databank* | 1124 | 1196 | 1322 | 1364 | 1559 | 1653 | 1739 | 1637 | 1806 | 1843 | Increase | Increase |  |
| *Norwegian Prescription Database data* | 1589 | 1833 | 1669 | 2136 | 1816 | 2396 | 1930 |  | 1984 |  | Increase | Increase |  |
| *Norwegian Wholesale data* | 1940 | 1833 | 2050 | 2136 | 2200 | 2396 | 2400 |  | 2420 |  | Increase | Increase |  |
| **Fentanyl** |  |  |  |  |  |  |  |  |  |  |  |  |  |
| *Netherlands GIP databank* | 1883 | 8701 | 2067 | 10960 | 1929 | 11675 | 1980 | 12302 | 2015 | 12579 | Increase | Increase | x |
| *Norwegian Prescription Database data* | 859 | 6358 | 858 | 6816 | 918 | 6733 | 894 |  | 875 |  | Increase | Increase | x |
| *Norwegian Wholesale data* | 1360 | 6358 | 1370 | 6816 | 1430 | 6733 | 1400 |  | 1350 |  | Increase, decrease | Increase, decrease |  |
| **Buprenorphine** |  |  |  |  |  |  |  |  |  |  |  |  |  |
| *Netherlands GIP databank* | 271 | 271 | 297 | 327 | 312 | 437 | 307 |  | 306 |  | Increase | Increase |  |
| *Norwegian Prescription Database data* | 451 | 2045 | 478 | 2487 | 502 | 2186 | 537 |  | 552 |  | Increase | Increase | x |
| *Norwegian Wholesale data* | 660 | 2045 | 700 | 2487 | 730 | 2186 | 780 |  | 770 |  | Increase | Increase |  |

ND = national database data

Norway (morphine, oxycodone, fentanyl, buprenorphine) and Netherlands (buprenorphine) INCB data: 2014 = data from 2012-2014; 2015 = data from 2014-2016; 2016 = data from 2016-2018
