# Supplementary material for: Is Europe facing an opioid crisis like the United States? An analysis of opioid use and related adverse effects in 19 European countries between 2010 and 2018
Source: Eur Psychiatry. 2021 Jun 21;64(1):e47. doi: 10.1192/j.eurpsy.2021.2219 (PMC8316471; doi:10.1192/j.eurpsy.2021.2219)
Supplement: Supplementary file 1 [file epasup.zip › S0924933821022197sup001.docx]

***Supplement***

***A. Notes on outcome 1: Prescription opioid consumption***

The yearly Technical Reports from the United Nations’ International Narcotics Control Board (INCB) were used to provide data on the national consumption of several different opioids in European countries and the US. Consumption is defined by the INCB as a drug that has been “supplied to any person or enterprise for retail distribution, medical use or scientific research”. Opioids distributed to pharmacies and hospitals are thus presented as “consumed”, even though some may, for example, stay in storage without being used that year. Assuming that the amount in stock is stable, this is not a serious limitation. Other potential limitations of our INCB data relate to the use of the ‘daily defined doses for statistical purposes’ (s-DDD), because (a) not all opioids have to be reported to the INCB (buprenorphine), (b) countries use different methods to collect their data, (c) certain opioids are used in different countries for different treatments in different doses, and (d) differences in clinical potency are not fully accounted for.^1,2^ Nevertheless, s-DDD can be regarded as a valid unit for the national consumption of prescribed opioids when used for within country comparisons over time and – if applied cautiously – for between country comparisons. To validate the INCB data, a set of data was requested on prescription opioid use from the Netherlands and Norway.

For the Netherlands, the GIP databank provided s-DDD data for drugs dispensed via prescription by physicians outside of hospitals and nursing homes in the Netherlands (data sourced from nearly all Dutch health insurance companies). For Norway, the Norwegian Prescription Database at the Norwegian Institute of Public Health provided s-DDD data for drugs dispensed by prescription outside of hospitals and nursing homes in Norway.^3^ In addition, the Norwegian Drug Consumption report from the Norwegian Institute of Public Health was used to provide s-DDD data based upon sales from all drug retailers in Norway.^4^ Both countries used the standard DDD as defined by the WHO to calculate s-DDD’s.^5^

The results are presented in Table 7. There were considerable differences between the national databases and the INCB data for fentanyl consumption reported in the Netherlands and Norway (higher consumption according to the INCB data), buprenorphine consumption reported in Norway (higher consumption according to the INCB data), and a trend for morphine consumption in the Netherlands between 2014 and 2018 in the GIP databank compared to the INCB data. Some of these data system differences were in line with previous findings of a comparison made in 2002 between data bases from five Nordic countries and the INCB.^6^ The most probably explanation for the disparity in s-DDD estimates is that INCB data is based upon import and export of opioids in weight, while the national data is based upon prescriptions filled by outpatients. This hypothesis is supported by the differences found between the two national databases in Norway; where one database recorded consumption based upon wholesales, and the other based upon drugs dispensed by prescription outside hospitals and nursing homes. The INCB fentanyl estimate is probably also larger because by calculating s-DDD’s based upon weight, the surplus fentanyl that remains in the transdermal application (fentanyl patch) after use is also counted as consumed. Finally, in contrast to the WHO, the INCB definition for s-DDD does not take into account different routes of opioid administration and thus uses as a single conversion factor per type of opioid. In practice this means that by only calculating DDD’s using the sublingual/nasal DDD for fentanyl of 0·6 mg, the transdermal form (DDD 1·2 mg) is not accurately represented in the INCB data.

***B. ICD Codes***^7^

ICD-10

F11 Mental and behavioural disorders due to opioids

F11·0 Mental and behavioural disorders due to opioids: Acute Intoxication

F11·1 Mental and behavioural disorders due to opioids: Harmful Use

F11·2 Mental and behavioural disorders due to opioids: Dependence Syndrome

T 40 Poisoning by narcotics and psychodysleptics (hallucinogens)

T40·0 Opium

T40·1 Heroin

T40·2 Other opioids

T40·3 Methadone

T40·4 Synthetic opioids

T40·6 Other and unspecified narcotics

X40-X44 Accidental poisoning

X60-X64 Intentional poisoning by drugs, medicaments and biological substances

Y10-Y14 Event of undetermined intent, poisoning

***C. Data sources per country outcomes 3-5***

Austria

All data was obtained from the annex of the 2019 *Addiction Epidemiology Report from Gesundheit Österreich.*^8^

Outcome 3: Hospital admission data (longer than 24 hours) for opioid misuse (ICD-10 codes F11·1 and F11·2) per 100,000 inhabitants (15-64 years old) was obtained from table 3. This data was from the Federal Ministry of Labour, Social Affairs and Consumer Protection (BMASKG). The authors report that the data prior to 2015 is likely to be an underestimation due to missing patient ID’s.

Outcome 4: Data on total direct drug related deaths (DRD) due to acute intoxication per 100,000 inhabitants (15-64 years old) was obtained from table 119 and using data on toxicology verified DRD by drug (percentage: heroin/morphine, methadone, or other opioids) (table 122), rates per opioid type were calculated. This data comes from multiple sources including BMASGK transmitted police reports, forensic data, and data from the General Mortality Register (based on ICD-10 codes) from Statistics Austria. Polydrug mortality cases were counted multiple times in the dataset.

Outcome 6: Yearly OST patient data per 100,000 inhabitants (15-64 years old) was obtained from tables 4 and 6.

Belgium

Outcome 3: Opioid hospital admissions data (rate per 100,000 population 15 years and older) was acquired from the Belgium Federal Public Service (Data and Strategic Information Department, FPS Public Health, Food Chain Safety and Environment, Belgium). From 2016 data was based on ICD-10 codes: T40·0-T40·4 and F11. Before this data was based on ICD-9 codes 96500 opium, 96501 heroin, 96502 methadone and 96509 other opioids. Data on diagnostics and procedures for the registration year 2015 was not available due to the transition from ICD-9-CM to ICD-10-BE.

Outcome 4: Total opioid mortality data (rate per 100,000 population 15 years and older) was acquired by Sciensano Belgium (Epidemiology and Public Health) using the data from the General Mortality Register in Belgium. It is known that this database underreports drug related cases. Opioid mortality was defined using the ICD-10 codes: F11 plus X42, X62, Y12 and T40·0-T40·4.

Outcome 5: Treatment admission data by primary drug (heroin, fentanyl, carfentanil, tramadol, oxycodone, opium, methadone and buprenorphine) (rate per 100,000 population 15 years and older) was acquired via contact with Sciensano Belgium (Epidemiology and Public Health) using data from the national addiction treatment register (TDI).^9^ This register follows the EMCDDA Treatment Demand Index (TDI) protocol Version 3·0 and compiles annual data from outpatient and inpatient services in specialised centres and hospitals.^10^ The register was started in 2011, however there was only stable data with good coverage available since 2015. The data concerns patients entering treatment for the first time ever or patients re-entering treatment after they stopped their previous treatment episode. A treatment episode was considered stopped if: (a) in the case of ambulatory treatment: the patient didn’t show up for 3 months; (b) in the case of residential treatment: the patient left the institution and no follow-up intake was foreseen.

Outcome 6: OST data was extracted from the EMCDDA *Belgium Country Drug Report 2019.* ^11^ Rates were calculate (18 years and older) using population data from Statbel.^12^

Cyprus

Outcome 4: Opioid overdose mortality data (all opioids and oxycodone) was acquired via personal contact with the Head of the Cyprus National Addictions Authority. Data is the number of cases and thus polydrug mortality is not counted multiple times in the dataset. Rates per 100,000 inhabitants (15 years and older) were calculated using data from the Republic of Cyprus Statistical Service.^13^

Outcomes 5 and 6: OST patient data (methadone and buprenorphine; oxycodone was not included, rates per 100,000 inhabitants based on the 2011 population census) and opioid treatment admissions data (rates per 100,000 inhabitants 15 years and older) were acquired via personal contact with the Head of the Cyprus National Addictions Authority. This register follows the EMCDDA TDI protocol Version 3·0.^10^

Czech Republic

Outcome 3: Data regarding hospitalisations (longer than 24 hours) for drug intoxication in emergency hospitals was obtained from the *Drug Situation in the Czech Republic in 2018* report (table 28).^14^ The data was sourced from the National Hospitalisations Registers, which records cases of hospital stays classified by ICD-10 codes (T40·0-T40·4). Rates were calculated per 100,000 inhabitants (15 years and older) using population data from the Czech Statistical Office.^15^

Outcome 4: Fatal opioid drug overdose data and toxicology of overdose deaths by type of opioid was collected from the yearly *Drug Situation in the Czech Republic* reports (2015-2018).^16^ The data comes from the Special Mortality Register which reports the findings of mandatory autopsies by a forensic medical examiner in all cases of violent deaths (i.e., injuries and poisoning) since 2015. Data presented is the sum of mono and polydrug deaths. Rates were calculated using the same method as for outcome 3.

Outcome 5: Data on treatment entrants by primary drug was collected from the data tables submitted to the EMCDDA (Characteristics of individuals starting treatment for drugs), acquired via personal contact with the Czech Republic EMCDDA partner. This data follows the EMCDDA TDI protocol Version 3·0.^10^ Data was only collected for the years 2014-2018 because of the new National Register which would not allow for a reliable comparison of trends before and after 2014. Data in 2014 and 2015 came from outpatient, inpatient and low-threshold treatment centres. In 2017 and 2018 there was also data from general practitioners and treatment units in prisons. Rates were calculated using the same method as for outcome 3.

Outcome 6: OST data was collected from the *Drug Situation in the Czech Republic in 2018* report (figure 29).^14^ The data collected after 2014 is an underestimation due to technical issues in the new National Register of Treatment of Drug Users resulting in a significant number of patients receiving OST that are not reported in the registry. While the data collected shows 2,312 patients in 2018, the authors estimate that there are around 5,000 patients receiving OST. Rates were calculated using the same method as for outcome 3.

The United Kingdom (UK)

Outcome 3 (England): Hospital admission data for poisoning by opioid type (ICD-10 codes T40.0-T40.4) and admissions for mental and behavioural disorders due to opioids (ICD-10 code F11) was extracted from the National Health Service (NHS) Statistics on Drug Misuse dataset.^17^ Rates per 100,000 inhabitants (15 years and older) were calculated using population data from the Office for National Statistics.^18^

Outcome 4 (England &Wales): The worksheet *Deaths related to drug poisoning by selected substances in England and Wales* (1993 to 2018), was used to obtain data on opioid mortality. This worksheet reports deaths when the underlying cause was drug poisoning, i.e., ICD 9 codes (292, 304, 305.2-305.9, E850-E858, E950.0-E950.5, E962.0, E98.0-E980.5) and ICD 10 codes (F11-F16, F18-F19, X40-X44, X60-X64, X85, Y10-Y14), and the specified substance was mentioned on the death certificate. Polydrug mortality cases were counted multiple times in the dataset. Rates were calculated using the same method as outcome 3.

Outcome 5 (England): Treatment entrants data by primary drug (all opioids, heroin, methadone, buprenorphine and other opioids) was sourced from the data tables in the *UK Drug Situation 2019* report (table 3·11) which follows the EMCDDA TDI protocol Version 3·0.^19^ Rates were calculated using the same method as for outcome 3.

Outcome 6 (UK):Yearly OST data was extracted from the EMCDDA *England Country Drug Report 2019.*^20^ Rates were calculated using the same method as for outcome 3.

Estonia

Outcome 4: Total opioid and fentanyl overdose mortality was acquired via personal contact with the Estonian EMCDDA partner who had sourced the data from the national register of cause of deaths from the National Institute for Health Development (NIHD). Rates per 100,000 inhabitants (15 years and older) were calculated using the population statistical database from Statistics Estonia.^21^

Outcome 5 and 6: Yearly treatment and OST data was also acquired via personal contact with the Estonian EMCDDA partner, who had extracted the data from the NIHD. Because there has only been a personalized drug treatment database since February 2020 there was no data available on total treatment by type of opioid. Rates were calculated using the same method as for outcome 4.

France

Outcome 3: Outcome 3 (England): Hospital admission data for poisoning by opioid type (ICD-10 codes T40.0-T40.4) and admissions for mental and behavioural disorders due to opioids (ICD-10 code F11) was acquired via personal contact with Dr Chouki Chenaf. Rates per 100,000 inhabitants (15 years and older) were calculated using population data from the French Institute for Demographic Studies (INED).^22^

Outcome 4: Drug overdose related deaths data was acquired via personal contact with the Head of Research at the French Monitoring Centre for Drugs and Drug Addictions. The data was sourced from the Drug and Substance Abuse-Related Deaths survey (DRAMES), coordinated by the National Agency for Medicines and Health Products Safety (ANSM) and the Network of the Regional Abuse and Dependence Monitoring Centres (CEIP-A) in Grenoble. For the years 2010, 2011 and 2013 there was no breakdown in the type of opioids responsible for deaths due to other medications than OST. In the other years there was a detailed analysis per type of opioid. Polydrug mortality cases were counted multiple times in the dataset. Rates were calculated using the same method as outcome 3.

Outcome 5: Data on the number of people starting treatment whereby opioids were the most problematic substance was acquired from the 2019 National report to the EMCDDA by the French Reitox National Focal point. Data was available from 2014 to 2018.^23^ It should be noted that the number of outpatient specialist treatment centres that provided data each year between 2014 and 2018 represents just over half of the total number of outpatient treatment centres. To eliminate variations related to changes in the scope of respondents/centres included, the data provided was analysed in constant terms, i.e., on a subset of treatment centres that reported each year between 2014 and 2018. Centres which did not provide data did not seem to display common characteristics which would distinguish them from those having submitted data. This data, however, is thus not a representation of all treatment admissions, and should be used to follow trends. Rates were calculated using the same method as outcome 3.

Outcome 6: OST data was acquired via personal contact with the Head of research at the French Monitoring Centre for Drugs and Drug Addictions. The data was sourced from Échantillon généraliste des bénéficiaires simplifié (EGBS, General sample of French persons with social security coverage), National public health insurance (CNAM), the French Monitoring Centre for Drugs and Drug Addiction (OFDT) and Csaspa activity reports (a national treatment and prevention centre for addiction). Rates were calculated using the same method as for outcome 3.

Germany

Outcome 3: Acute poisoning hospital admissions data for opioids (defined by ICD-10 codes T40.0-T40.3 and F11.0) was acquired via personal contact with the German Monitoring Centre for Drugs and Drug Addiction. The data was sourced from the *Statistical Report on Hospital Diagnoses* from the German Federal Statistical Office (FSO). Rates per 100,000 inhabitants (entire population) were calculated using population data from the FSO.^24^

Outcome 4: Opioid mortality data was acquired via personal contact with the German Monitoring Centre for Drugs and Drug Addiction. Two separate systems for recording drug deaths were used to provide two datasets. Firstly, the *Statistical Report on the causes of death* (General Mortality Register) from the German Federal Statistical Office was used to provide data on total opioid-related deaths (with opioids in the ICD-10 X or Y code) for comparison with other European countries. Secondly, the police data from the Drugs Data File (collected by the Federal Criminal Police Office BKA) was used to provide data on mono and polydrug poisonings by type of opioid (heroin/morphine, methadone/polamidone, buprenorphine, other OST drugs, fentanyl and synthetics opioids [fentanyl derivatives]). The mono and poly poisoning data was added together and polydrug mortality cases were counted multiple times in the dataset. This data shows long-term secondary diseases, suicides and accidents that have come to the attention of the police and was included because it gives insight into the specific type of opioids that are involved in overdose mortality in Germany. The data is, however, less suitable for European comparisons due to differences in selection criteria and reported age groups. Rates were calculated using the same method as for outcome 3.

Outcome 5: Treatment admissions data for opioid related problems (defined by ICD-10 code F11) was obtained via personal contact with the German Monitoring Centre for Drugs and Addiction, who had sourced their data from the *Statistical Report on Substance Abuse Treatment in Germany.*^25^ The data is based upon specialized addiction care services, covering both outpatient and inpatient treatment. It should be noted, however, that with respect to inpatient care many larger facilities, which also offer addiction specific treatments, are not represented in the data. Furthermore, for the years 2002-2004 data presented only refers to outpatient treatment. Rates were calculated using the same method as outcome 3.

Outcome 6: Data on the number of registered OST patients was obtained from the 2019 *Drugs and Addiction Report* (figure 45) and supported by personal contact with the German Monitoring Centre for Drugs and Drug Addiction, who had sourced their data from the *Report on the Substitution Register* by the Bundesopiumstelle (BOPST).^26^ Rates were calculated using the same method as for outcome 3.

Ireland

Outcome 3: Data on non-fatal opioid overdose patients discharged from Irish hospitals was obtained from the yearly *Focal Point Ireland national reports (harm and harm reduction*) based on the Hospital In-Patient Enquiry (HIPE) scheme data.^27^ Rates per 100,000 inhabitants (15 years and older) were calculated using population data from the Irish Central Statistics Office.^28^

Outcome 4: The number of opioid poisoning deaths (all opioids, methadone, heroin and fentanyl) was obtained from the 2017 *Health Research Board Bulletin*, and tramadol poisoning mortality data was acquired via personal contact with the Irish Health Research Board (HRB).^29^ All data was sourced from the Irish National Drug-Related Deaths Index (NDRDI). The NDRDI records data from four sources: The Coroner Service, the Hospital In-Patient Enquiry scheme (HIPE), the Central Treatment List (CTL), and the General Mortality Register (GMR) in order to ensure that the database is complete and accurate. Cases from the different data sources are cross matched on a selection of variables, including name, gender, county of residence, date of birth and date of death. This allows the NDRDI to eliminate duplicates and to maximise the amount of information available on each case recorded on the database. More detailed information on the methodology can be found in the previously published Health Research Board Trends Series papers. Polydrug mortality cases were counted multiple times in the dataset. Less than 5 deaths were presented as “0” in the data. Rates were calculated using the same method as outcome 3.

Outcome 5: Drug treatment entrants data (never previously treated, previously treated and unknown) for all opioids, heroin (and diamorphine), methadone (prescribed, street and unspecified), buprenorphine (prescribed and street), fentanyl, oxycodone, tramadol and ‘any other’ was acquired from the National Drug Treatment Reporting System Data from the HRB National Drugs Library.^30^ The dataset includes cases treated in all types of services: outpatient, inpatient, GPs, low threshold (providing low-dose methadone or drop-in facilities only), and those treated in prison. The dataset is case based which means that there is a possibility that individuals are counted more than once in the database, for example when a person receives treatment at the same center more than once per calendar year. Rates were calculated using the same method as outcome 3.

Outcome 6: OST data was sourced from the EMCDDA *Ireland Country Drug Report 2019.*^31^ The data excludes a small number of patients receiving OST with buprenorphine/naloxone. Rates were calculated using the same method as outcome 3.

Italy

Outcome 3: Hospital admissions data related to opioids (ICD 9 codes: 3040, 3055, 9650) was acquired via personal contact with the EMCDDA partner in Italy. Rates per 100,000 inhabitants (15 years and older) were calculated using population data from the Italian National Institution for Statistics (for the years 2000/01 the population from 2002 was used).^32^

Outcome 4: The number of fatal substance related overdoses per type of opioid was acquired via personal contact with the EMCDDA partner in Italy. The data was acquired from the Special Registry which collects the deaths directly linked to drug use (acute poisoning, overdose) and reported by the Police Forces and Prefectures to the Central Directorate for Anti-Drug Services of the Ministry of the Interior. Data presented is number of cases thus polydrug mortality is not counted multiple times in the dataset. Rates were calculated using the same method used for outcome 3.

Outcome 5 and 6: OST patient data and outpatient opioid addiction treatment admissions by primary drug (heroin, methadone, buprenorphine, other opioids) was acquired via personal contact with the EMCDDA representative in Italy. In 2015 a treatment was considered established when the patient was provided at least two health care sessions during the reference period, however after 2015 it was considered established after at least one health care session. Rates were calculated using the same method as for outcome 3.

Latvia

Outcome 3: Data on the number of opioid-related hospital inpatient treatment entrants (defined by primary diagnose ICD-10 F11) was acquired via personal contact with the Centre for Disease Prevention and Control Republic of Latvia, who had sourced their data from the National Health Service Registry of Inpatient Treatment Services (SPANS). If a patient was admitted for multiple opioid-related episodes, it was only included once in the data set. It should be noted that the data only covers state funded health services, and thus not the private institutions, however most inpatient services are covered by the state. Rates per 100,000 inhabitants (15 years and older) were calculated using population data from Statistics Latvia.^33^

Outcome 4: Opioid-related deaths data was also acquired via personal contact with the Centre for Disease Prevention and Control Latvia, who had sourced their data from the National Causes of Death Database (defined by ICD-10 codes X41, X42, X44 ,X61, X62, X64, Y11, Y12, Y14 with a combination of T40·0-T40·4). Polydrug mortality was counted multiple times in the data set. There were several under reporting issues. Firstly, there are many more ill-defined causes of deaths for young adults in Latvia, for example due to cardiovascular problems, and it is unknown how this influences the results. Secondly, fluctuations in funding influence the number of deceased being sent to forensic laboratories. Finally, due to limited technical possibilities the forensic laboratories were sometimes not able to assess which drugs were involved in mortality (especially new psychoactive substances). Operational information from the police confirms that there are deaths from carfentanil and other synthetic opioids, but in the data provided it was not detected. Rates were calculated using the same method as outcome 3.

Outcome 5 and 6: Data on number of patients who received State funded drug treatment services in a calendar year with primary diagnosis ICD-10 F11 and number of clients in OST were sourced via personal contact with the Centre for Disease Prevention and Control Latvia. Rates were calculated using the same method as outcome 3.

Lithuania

Outcome 3 and 4: Hospital admission data for non-fatal opioid overdoses and drug related deaths (both outcomes organised by ICD-10 T40·0-T40·4, mortality data in combination with X42 and Y12) was obtained via personal contact with the National Focal Point of Lithuania. Rates per 100,000 population (15 years and older) were calculated using population data from the Official Statistics Portal from Statistics Lithuania.^34^

Outcome 5: Treatment admissions data was also obtained via personal contact with the National Focus Point of Lithuania. Data was collected following the definitions used in the EMCDDA TDI protocol version 3·0 from the Lithuanian treatment system called ASIS, however there were some limitations to the data coverage. Rates were calculated using the same method as outcome 3.

Outcome 6: Data on the number of OST clients in treatment on the 1^st^ of January every year was also obtained via personal contact with the National Focal Point of Lithuania and rates were calculated using the same method as outcome 3.

Northern Ireland

Outcome 3: Non-fatal opioid related hospital admissions data (defined by ICD-10 codes T40·0-T40·4, F11 and F11.0) was acquired via contact with Statistics Northern Ireland, who had sourced their data from the Northern Ireland Hospital Inpatient System (Hospital Information Branch, Information & Analysis Directorate, Department of Health) and calculated rates per 100,000 inhabitants (15 years and older) using mid-year population estimates from the Northern Ireland Statistics and Research Agency (NISRA).^36^ They stated that deaths and discharges were used as an approximation of admissions and the figures should not be used to denote individuals as a person may be admitted more than once in the same period. If there were less than 5 people admitted in a group, then it was counted as “0” in the data set to protect patient confidentiality.

Outcome 4: Opioid related deaths data (all opioids, heroin/morphine, methadone, tramadol, oxycodone and fentanyl) was extracted from the NISRA *Drug Related and Drug Misuse Deaths 2007-2017* data tables.^35^ Polydrug mortality cases were counted multiple times in the dataset. Rates were calculated using the same method as outcome 3.

Outcome 5 and 6: OST data was obtained from the *Northern Ireland Substitute Prescribing Database Report 31 March 2018.*^37^ Data on treatment admissions to drug addiction services by main drug was obtained from the *Northern Ireland Substance Misuse Database* annual reports.^38,39^ A 6 month rule was used in the data to remove duplicates where a single individual is reported multiple times (i.e., if the same patient presented to treatment multiple times within a 6 month period they were counted as a single record). If a patient presented to treatment after 6 months from the date of initial first face to face they were recorded as a separate record, and while this methodology affords the potential for a client to be recorded twice in a given year, the numbers seen for such multiples are small (e.g., 33 clients in 2016/2017). Data was reported in financial years as opposed to calendar years and thus the data presented in table 2 in the yearly column ‘2010’ is from the database years 2010/2011 and so forth. Rates were calculated using the same method as outcome 3.

The Netherlands

Outcome 3: Hospital admission data for opioid intoxications (ICD 10 codes T40·0-T40·4) was sourced from the Dutch National Hospital Care Basic Registration (Landelijke Basisregistratie Ziekenhuiszorg), a database managed by Dutch Hospital Data. This database contains medical, administrative, and financial information on hospital admissions and covers all Dutch hospitals.^40^ Rates (15 years and older) were calculated using population data from StatLine.^41^

Outcome 4: Opioid mortality data (all opioids, ICD-10 codes T40·0-T40·4) was sourced from the Statistics Netherlands (CBS), and came from cause-of-death statistics databases, which contains information on the deaths of all inhabitants. After a death occurs, a physician or pathologist is required to fill out a cause-of-death form for statistical purposes. Rates were calculated using the same method as outcome 3.

Outcome 5 and 6: Opioid treatment admissions data, following the EMCDDA TDI protocol Version 3·0, and OST data was sourced from the Dutch National Alcohol and Drugs Information System (LADIS). It is important to note that some treatment centres record “methadone” as the primary problem for patients using methadone for OST, which could result in an overestimation of methadone treatment admissions. Furthermore, some patients do not have a primary problem recorded in the system but are receiving methadone treatment and are thus not represented in the treatment admissions analysis per opioid (e.g., 22 patients in 2018). These patients are, however, represented in the total opioid treatment analysis and OST analysis. Rates were calculated using the same method as outcome 3.

Norway

Outcome 4: Opioid mortality data was acquired from the Norwegian Cause of Death Registry from the Norwegian Institute for Public Health (defined by ICD-10 codes T40.0-T40.4 and T40.6 where the underlying cause of death was one of the following codes X42, X44, X62, Y12 and Y14). Rates per 100,000 inhabitants 15 years and older were calculated using population data from the Norwegian Institute for Public Health.

Outcome 6: OST data was acquired from the EMCDDA yearly country report.^42^ Rates were calculated using the same method as outcome 3.

Russia

Outcome 4: Data on the number of patients hospitalized in psychiatric hospitals for opioid addiction (per 100,000 general population) in the Russian Federation was acquired via personal contact with the Vice Director for Research and Head of the Department of Addictions at the Bekhterev National Medical Research Centre for Psychiatry and Neurology in St Petersburg Russia, who had sourced his data from two statistical compendiums.^43,44^

Scotland

Outcome 3: General acute and psychiatric hospital admission data for opioid overdoses (ICD-10 codes T40·0-T40·4) and mental and behavioural disorders due to opioids (ICD-10 code F11) was extracted using the data explorer Drug-Related Hospital Statistics on Drug and Alcohol Misuse and contact with Public Health Scotland (data source was the Scottish Drug Misuse Database: SDMD).^45^ Data presented in table 2 in the yearly column ‘2010’ is from the database years 2010/2011 and so forth. Rates per 100,000 inhabitants for ICD-10 codes T40·0-T40·4 were extracted. For the codes T40·2 and T40·4 admissions were only included in the data set if it was combined with an ICD-10 mental and behavioural substance misuse disorder code (F11) within the same hospital stay. This was done by the statisticians because they believe that because these drug types are widely prescribed, the overdose as a results of problematic drug use can only be reliably determined where other evidence of problematic drug use is included in the hospital record. Although this additional validation measure enhances the validity of the figures, it may also produce an underestimation of overdoses associated with problematic use of these substances. Data are presented in European age-sex standardised rates per 100,000 population (for more information please see Appendix 1 in the full report *Drug Related Hospital Statistics Scotland 2017/18*)*.*^46^

Outcome 4: Opioid mortality data (total opioid mortality, heroin, methadone, buprenorphine and tramadol) was acquired from the *Drug-related Deaths in Scotland 2018* data tables published by the National Records of Scotland (NRS), Table Y: Drug-related deaths, on the basis of the Office for National Statistics (ONS) ‘wide’ definition.^47^ Compared to the standard definitions, the ‘wide’ definition also includes deaths due to opioids/opiates in compound analgesics and cold remedies. These are deaths coded with ICD10 codes F11 in combination with X40-44, X0-64, X85 and Y10-Y14. If more than one drug was reported, cases were reported multiple times in the dataset. Data on fentanyl was obtained from the extra tables that cover ‘less common drugs’ (Table SUB1). Numbers represent selected drugs that were considered by the pathologist as implicated in or which potentially contributed to the cause of death. Rates were calculated per 100,000 inhabitants (15 years and older) using population data from the NRS.^48^

Outcome 5: Data on the main illicit drug use by primary assessment for treatment (medical services such as general practice, hospital etc. and specialist drug services) was extracted from table 3·2 (Drug use Profile- main drug, number of individuals in SDMD database reporting illicit drug use by main illicit drug) from the SDMD 2018/19 data tables.^49^ Extra data on the breakdown of other opioids from the SDMD tables on main illicit drug during primary assessment for treatment (into fentanyl/oxycodone, morphine sulphate tablets, codeine (unspecified), opioid containing analgesics, tramadol and other remaining opioids) was requested from Public Health Scotland. Rates were calculated using the same method as outcome 4. If an individual had more than one initial assessment in each year, only the first assessment record was included. There was no data available for the years 2012 and 2013 due to data quality issues. Some data was also not available due to suppression given the potential risk of disclosure and to help maintain patient confidentiality. It should be noted that the data presented does not reflect the total number of individuals treated because it is recorded during the assessment for treatment and some early drop-outs (estimated at 8%) may not actually receive treatment. Public Health Scotland stated that the completeness of the SDMD data for the years 2014/15-2018/19 was 71·8%, 66·7%, 67·0%, 64·6%, and 59·6% respectively compared to the Drug and Alcohol Treatment Waiting Times database (DATWT). These are separate systems which are managed separately locally and have different processes and procedures. While these completeness estimates may be inexact due to contextual and system-related issues, the extent of the differences observed in recording between the DATWT database and the SDMD means that it is not appropriate to consider individuals recorded on SDMD as the complete population of individuals assessed for specialist drug treatment.

Outcome 6: OST data was estimated using the *Methadone Patient Analysis* from the Public Health Information for Scotland.^50^ This is an estimation of the number of patients in OST using the Community Health Index (CHI) numbers captured on prescriptions. This data should be interpreted with caution because issues associated with CHI capture for methadone prescriptions make it impossible to provide a robust count of the number of individuals prescribed methadone as an OST in Scotland. Rates were calculated using the same method as outcome 4.

Slovakia

Outcome 3: Opioid related hospital admissions data (ICD-10 codes F11 and T40) was obtained via personal contact with the Slovak National Health Information Centre (Department for Media and Public Relation) who sourced the data from the Statistical Office of the Slovak Republic. Rates per 100,000 inhabitants (15 years and older) were calculated using data from the Statistical Office of the Slovak Republic.^51^

Outcome 4: Opioid mortality data defined by ICD-10 codes F11 or X42, X62 and Y12 during the period 2010 to 2018 was acquired via personal contact with the Slovak National Health Information Centre (Department for Media and Public Relation) who sourced the data from the Statistical Office of the Slovak Republic. Rates were calculated using the same method as outcome 3.

Outcome 5: Data regarding the number of drug dependent people treated in Slovakia (outpatient, inpatient, hospitals, health care centres, prison health care departments etc) was acquired via personal contact with the Coordinator of the Slovak Early Warning System (EWS) at the National Monitoring Centre for Drugs (Ministry of Health of the Slovak Republic). Data was calculated following the EMCDDA TDI protocol version 3·0, however the allocation of certain drugs into groups did not follow the protocol. Rates were calculated using the same method as outcome 3.

Outcome 6: OST data was acquired from the EMCDDA *Slovakia Country Drug Report 2019.*^52^ Rates were calculated using the same method as outcome 3*.*

Switzerland

Outcome 3: Opioid hospital admissions data (ICD-10 codes T40·0-T40·4, F11) was sourced from the Federal Statistics Office (FSO) data tables.^53^ Rates per 100,000 inhabitants (15 years and older) were calculated using population data from the FSO.^54^

Outcome 5 and 6: Data on the number of patients receiving OST for heroin addiction between was obtained from the *National Substitution Statistics* website. The data was sourced from *act-info* - a uniform, Swiss-wide client monitoring system for addiction support in outpatient and inpatient centres.^55^ Rates were calculated using the same method as outcome 3. Data on the main problem drug when entering treatment in Switzerland was sourced from the yearly *act-info* reports.^56^ Rates were calculated using the same method as outcome 3. It should be noted, however, that this data is an underestimation because it is based on participating centres only and not all existing treatment facilities. The annual recorded patient numbers are therefore not a true reflection of the number of patients in treatment, nor a reflection of trends in treatment since they are yearly affected by different participating treatment centres. Furthermore, the recorded patient numbers may lead to a misinterpretation, due to growing registered numbers because of better registration systems, while in fact, cues from the field suggest a true trend in the opposite direction (a decrease in patients treated for opioid addictions). In the 2018 annual *act-info* report, the authors attempted to address this problem by only using data from centres that had delivered data in a constant way over the last years and using the shape of the curves (and not the actual numbers behind the graphs) to comment on trends in time for patients receiving treatment (graph 13 and 14).^57^ Opioids were included in this analysis, however numbers were a underestimation because patients receiving OST could not be included due to technical issues. These graphs showed that total treatment demand for opioids slightly increased between 2013 and 2014, with a downward trend since then (index 2013-2018: -22·3%). Regarding first time/new treatment admissions for opioid problems a declining trend was also seen (index: 2013-2018: -62·2%).

The US

Outcome 3: Data regarding the national rate (per 100,000 population) of opioid-related hospital admissions was extracted from the Healthcare Cost and Utilization Project (HCUP) Fast Stats. Data was defined by ICD-10 codes from October 1 2015: F11 series (except F11·11 and F11·21) and T40 series, and prior to this by ICD-9 codes: 304·00-304·02, 304·70-304·72, 305·50-305·52, 965·00-965·02, 965·09, 970·1, E850·0-E850·2, E935·0-E935·2, E940·1.^58^ Due to the changing coding systems trend line comparisons from 2014 to 2015 may be unreliable. The data only covers state funded health services (e.g. not private), however most inpatient services are covered by the state.

Outcome 4: Data on age-adjusted drug overdose death rates per 100,000 standard population (all opioids, heroin, natural and semisynthetic opioids (such as morphine, oxycodone and hydrocodone), methadone, synthetic opioids other than methadone (such as fentanyl, fentanyl analogues and tramadol)) was extracted from the data tables from the National Centre for Health Statistics Data Brief.^59^ Opioid mortality was defined by ICD-10 codes drug-poisoning (overdose: X40-X44, X60-X64, X85, Y10-Y14) in combination with opioids (T40·0-T40·4, T40·6). Polydrug mortality cases were counted multiple times in the dataset.

Outcome 5: Opioid treatment admissions data (all opioids, heroin, methadone, and for 2017 buprenorphine, oxycodone and tramadol) was obtained via the Drug & Alcohol Services Information System *Treatment Episode Data Sets* Annual Reports.^60,61^ Rates per 100,000 population were calculated using population data from the United States Census Bureau.^62^

Outcome 6: OST treatment data (buprenorphine, injectable naltrexone or methadone) was extracted from the yearly reports from the National Survey of Substance Abuse Treatment (N-SSATS).^63^ This dataset is an underestimate of the total number of patients receiving OST because patients prescribed buprenorphine through an independent DATA 2000-waiverd medical practitioner or naltrexone through an independent practitioner not affiliated with a substance abuse treatment facility are not represented in the dataset. Rates were calculated using the same method as outcome 5.

***D. Definitions Outcome 3 “All opioid related admissions”***

England, France, Northern Ireland, Scotland, Slovakia, Switzerland, and the US: ICD10 codes F11 and T40·0-T40·4

Belgium: Only T40·0-T40·4 up to and including 2014, hereafter also including F11

Germany: T40·0-T40·4 and F11·0

The Czech Republic, The Netherlands and Lithuania: Only T40·0-T40·4

Austria: Only F11·1 and F11·2

Latvia: Only F11

Ireland: non-fatal opioid overdose admissions, not defined by ICD-10 code

Italy: total opioid related admissions, not defined by ICD-10 code

**References**

1. Svendsen K, Borchgrevink PC, Fredheim O, Hamunen K, Mellbye A, Dale O. Choosing the unit of measurement counts: The use of oral morphine equivalents in studies of opioid consumption is a useful addition to defined daily doses. Palliat Med. 2011 Mar 4;25(7):725–32.

2. Nielsen S, Gisev N, Bruno R, Hall W, Cohen M, Larance B, et al. Defined daily doses (DDD) do not accurately reflect opioid doses used in contemporary chronic pain treatment. Pharmacoepidemiol Drug Saf. 2017 May;26(5):587–91.

3. Norwegian Institute of Public Health. Norwegian Prescription Database [Internet]. 2020 [cited 2020 Jun 12]. Available from: http://www.norpd.no/kontaktOss.aspx

4. Sakshaug S, Olsen K, Berg C, Blix HS, Dansie LS, Litleskare I, et al. Drug Consumption in Norway 2014-2018. Oslo; 2019.

5. WHO Collaborating Centre for Drug Statistics Methdology. ATC/DDD Index 2020 [Internet]. 2020 [cited 2020 May 10]. Available from: https://www.whocc.no/atc_ddd_index/

6. Hamunen K, Laitinen-Parkkonen P, Paakkari P, Breivik H, Gordh T, Jensen NH, et al. What do different databases tell about the use of opioids in seven European countries in 2002? Eur J Pain. 2008 Aug;12(6):705–15.

7. World Health Organisation. ICD-10 Browser [Internet]. [cited 2020 Jul 16]. Available from: https://icd.who.int/browse10/2019/en

8. Anzenberger J, Busch M, Grabenhofer-Eggerth A, Hojni M, Klein C, Schmutterer I, et al. Epidemiologiebericht Sucht 2019 Illegale Drogen , Alkohol und Tabak Annex. Vienna; 2019.

9. Antoine J, De Ridder K, Plettinckx E, Blanckaert P, Gremeaux L. Treatment for substance use disorders: the Belgian Treatment Demand Indicator registration protocol. Arch Public Heal. 2016;74(1):27.

10. European Monitoring Centre for Drugs and Drug Addiction. Treatment Demand Indicator (TDI) Standard protocol 3.0. Luxembourg; 2012.

11. European Monitoring Centre for Drugs and Drug Addiction. Belgium Country Drug Report 2019. Lisbon; 2019.

12. Statbel. Structure of the Population [Internet]. 2020 [cited 2020 Jun 18]. Available from: https://statbel.fgov.be/en/themes/population/structure-population#panel-13

13. Republic of Cyprus Statistical Service. Cyprus Population Summary Data [Internet]. 2020 [cited 2020 Mar 5]. Available from: http://www.cystat.gov.cy/mof/cystat/statistics.nsf/populationcondition_21main_en/populationcondition_21main_en?OpenForm&sub=1&sel=2

14. Mravčík V, Chomynová P, Grohmannová K, Janíková B, Černíková T, Fidesová H. Drug situation in the Czech Republic in 2018. Prague; 2020.

15. Czech Statistical Office. Czech Demographic Handbook 2018: Population by main age group and sex: 1920–2018 (1 July) [Internet]. [cited 2020 Apr 1]. Available from: https://www.czso.cz/csu/czso/czech-demographic-handbook

16. National Monitoring Centre for Drugs and Addiction. Drug situation in the Czech Republic 2015-2018. Prague; 2018.

17. National Health Service England. Drug related hospital admissions: data tables [Internet]. 2019 [cited 2020 Apr 1]. Available from: https://digital.nhs.uk/data-and-information/publications/statistical/statistics-on-drug-misuse/2019/drug-admissions-data-tables

18. Office for National Statistics. England Population mid year estimates [Internet]. [cited 2020 Apr 20]. Available from: https://www.ons.gov.uk/peoplepopulationandcommunity/populationandmigration/populationestimates

19. Public Health England. United Kingdom Drug Situation 2019: UK Focal Point On Drugs [Internet]. London; 2020 [cited 2020 Jul 29]. Available from: https://www.gov.uk/government/publications/united-kingdom-drug-situation-focal-point-annual-report/united-kingdom-drug-situation-focal-point-annual-report-2019

20. European Monitoring Centre for Drugs and Drug Addiction. United Kingdom Country Drug Report 2019: Treatment. Lisbon; 2019.

21. Statistics Estonia. Statistical Database: Population [Internet]. [cited 2020 May 23]. Available from: http://andmebaas.stat.ee/?lang=en#

22. French Institute for Demographic Studies. French Population by age [Internet]. [cited 2020 Apr 5]. Available from: https://www.ined.fr/en/everything_about_population/data/france/population-structure/population-age/#r153

23. D’Arleux JM, Lermenier-Jeannet A, de l’Eprevier A, Ades JE. 2019 National Report (2018 data) to the EMCDDA by the French Reitox National Focal Point. 2019.

24. Statistisches Bundesamt (Destatis). Population by age groups [Internet]. 2020 [cited 2020 Mar 12]. Available from: https://www.destatis.de/EN/Themes/Society-Environment/Population/Current-Population/Tables/lrbev01.html

25. Deutsche Suchthilfestastik. Statistical Report on Substance Abuse Treatment [Internet]. 2018. Available from: https://www.suchthilfestatistik.de/

26. Die Drogenbeauftragte der Bundesregierung. Drogen- und Suchtbericht 2019 [Internet]. Berlin; 2019. Available from: https://www.drogenbeauftragte.de/fileadmin/dateien-dba/Drogenbeauftragte/4_Presse/1_Pressemitteilungen/2019/2019_IV.Q/DSB_2019_mj_barr.pdf

27. Health Research Board. Irish National Focal Point to the European Monitoring Centre for Drugs and Drug Addiction. Focal Point Ireland: national report for 2019 – harms and harm reduction. [Internet]. Dublin; 2020. Available from: https://www.drugsandalcohol.ie/25259/

28. Central Statistics Office Ireland. Population and Migration Estimates [Internet]. 2019 [cited 2020 Apr 1]. Available from: https://www.cso.ie/en/releasesandpublications/er/pme/populationandmigrationestimatesapril2019/

29. Irish Health Research Board. National Drug‐Related Deaths Index 2008 to 2017 data. 2019.

30. HRB National Drugs Library. National Drug Treatment Reporting System (NDTRS) online interactive tables [Internet]. 2019 [cited 2020 Jun 22]. Available from: www.drugsandalcohol.ie/tables%0A%0AContact

31. European Monitoring Centre for Drugs and Drug Addiction. Ireland Country Drug Report 2019: Treatment. Lisbon; 2019.

32. Istituto Nazionale di Statistica. Population and Households Italy [Internet]. [cited 2020 May 6]. Available from: https://www.istat.it/en/population-and-households?data-and-indicators

33. Statistics Latvia. CBS Database: Population Statistics [Internet]. [cited 2020 Jun 6]. Available from: https://www.csb.gov.lv/en/statistika/db

34. Statistics Lithuania. Official Statistics Portal, Population data [Internet]. 2018. [cited 2020 May 28]. Available from: https://osp.stat.gov.lt/gyventojai1

35. Northern Ireland Statistics and Research Agency (NISRA). Drug Related and Drug Misuse Deaths 2007- 2017 [Internet]. 2019 [cited 2020 May 20]. Available from: https://www.nisra.gov.uk/publications/drug-related-and-drug-misuse-deaths-2007-2017

36. Northern Ireland Statistics and Research Agency (NISRA). 2019 Mid Year Population Estimates for Northern Ireland. 2020.

37. Public Health Agency (HSC). Northern Ireland Substitute Prescribing Database Report 31st March 2018 [Internet]. 2018. Available from: https://www.publichealth.hscni.net/publications/northern-ireland-substitute-prescribing-database-report-31-march-2018

38. Northern Ireland Department of Health. Statistics from the Northern Ireland substance misuse database 2016/17 [Internet]. 2018 [cited 2020 May 28]. Available from: https://www.health-ni.gov.uk/publications/statistics-northern-ireland-substance-misuse-database-201617

39. Northern Ireland Department of Health. Statistics from the Northern Ireland drug misuse database 2001/02 to 2015/16 [Internet]. 2016 [cited 2020 May 28]. Available from: https://www.health-ni.gov.uk/publications/statistics-northern-ireland-drug-misuse-database-200102-201516

40. Dutch Hospital Data. Landelijke Basisregistratie Ziekenhuiszorg (LBZ).

41. StatLine. StatLine: Population; sex, age and nationality, 1 January in the Netherlands. 2020.

42. European Monitoring Centre for Drugs and Drug Addiction. Norway Country Drug Report 2019. Lisbon; 2019.

43. Kirzhanova V, Grigorova N, Kirzhanov V, Sidoruk O. Key performance indicators of addiction treatment services in Russian Federation in 2015-2016. In: Statistical compendium, M: NSC of narcology. Serbsky Federal Medical Research Centre of Psychiatry and Narcology of the Ministry of Health of the Russian Federation; 2017. p. 183.

44. Kirzhanova V, Grigorova N, Kirzhanov V, Sidoruk O. Key performance indicators of addiction treatment services in Russian Federation in 2014-2015. In: Statistical compendium, M: NSC of Narcology. V. Serbsky Federal Medical Research Centre of Psychiatry and Narcology of the Ministry of Health of the Russian Federation; 2016. p. 183.

45. ISD and NHS Scotland. Drug Related Hospital Statistics [Internet]. 2019 [cited 2020 May 6]. Available from: https://www.isdscotland.org/Health-Topics/Drugs-and-Alcohol-Misuse/Publications/2019-05-28/data-explorer.html

46. National Services Scotland. Drug-related Hospital Statistics Scotland 2017/2018 [Internet]. 2019. Available from: https://www.isdscotland.org/Health-Topics/Drugs-and-Alcohol-Misuse/Publications/2019-05-28/2019-05-28-DRHS-Report.pdf

47. National Records of Scotland. Drug-related Deaths in Scotland in 2018 [Internet]. 2018 [cited 2020 Apr 12]. Available from: https://www.nrscotland.gov.uk/statistics-and-data/statistics/statistics-by-theme/vital-events/deaths/drug-related-deaths-in-scotland/2018/list-of-tables-and-figures

48. National Records of Scotland. Mid-Year Population Estimates [Internet]. 2020 [cited 2020 Apr 12]. Available from: https://www.nrscotland.gov.uk/statistics-and-data/statistics/statistics-by-theme/population/population-estimates/mid-year-population-estimates/population-estimates-time-series-data

49. National Statistics. Scottish Drug Misuse Database, data tables [Internet]. 2020 [cited 2020 Apr 12]. Available from: https://beta.isdscotland.org/find-publications-and-data/lifestyle-and-behaviours/substance-use/scottish-drug-misuse-database/

50. The Scottish Public Heath Observatory. Drug misuse: Treatment for drug misuse; Methadone Patient Estimates - Community [Internet]. 2018 [cited 2020 Apr 24]. Available from: https://www.scotpho.org.uk/behaviour/drugs/data/treatment-for-drug-misuse/

51. Statistical Office of the Slovak Republic. Population and migration data (STATdat). 2019.

52. European Monitoring Centre for Drugs and Drug Addiction. Slovakia Country Drug Report 2019. Lisbon; 2019.

53. Swiss Federal Statistical Office. Medizinische Statistik der Krankenhäuser:Anzahl Fälle und durchschnittliche Aufenthaltsdauer (DAD) nach Altersklasse und Diagnosekode [Internet]. [cited 2020 Apr 17]. Available from: https://www.bfs.admin.ch/bfs/de/home/statistiken/kataloge-datenbanken/tabellen.html?dyn_publishingyearend=2020&dyn_title=Anzahl Fälle und durchschnittliche Aufenthaltsdauer (DAD) nach Altersklasse und Diagnosekode

54. Swiss Federal Statistical Office. Population: Demographic balance by age [Internet]. 2020 [cited 2020 Apr 17]. Available from: https://www.bfs.admin.ch/bfs/en/home/statistics/catalogues-databases/data.assetdetail.9566510.html

55. Sucht Schweiz. Offizielle Website der nationalen Substitutionsstatistik [Internet]. 2020. [cited 2020 Mar 12]. Available from: https://www.substitution.ch/de/jahrliche_statistik.html&year=2000&trend

56. Schweizerische Eidgenossenschaft. act-info Jahresbericht 2004-2018 [Internet]. [cited 2020 Apr 6]. Available from: https://www.suchtschweiz.ch/forschung/act-info/act-info-sambad/

57. Schweizerische Eidgenossenschaft. act-info Jahresbericht 2018 [Internet]. 2018 [cited 2020 Apr 6]. Available from: https://www.suchtschweiz.ch/fileadmin/user_upload/DocUpload/BAG_act-info-2018_D.pdf

58. Agency for Healthcare Research and Quality. HCUP Fast Stats website, Opioid-Related Hospital Use path [Internet]. [cited 2020 May 27]. Available from: https://www.hcup-us.ahrq.gov/faststats/OpioidUseServlet

59. Hedegaard H, Minino A, Warner M. Drug overdose deaths in the United States 1999-2018. NCHS Data Brief, no 365. Hyattsville, MD; 2020.

60. Substance Abuse and Mental Health Services Administration (Center for Behavioural Health Statistics and Quality). Treatment Episode Data Set (TEDS): 2016. Admissions to and Discharges from Publicly Funded Substance Use Treatment. Rockville, MD; 2018.

61. Substance Abuse and Mental Health Services Administration (Center for Behavioural Health Statistics and Quality). Treatment Data Set (TEDS): 2017. Admissions to and Discharges from Publicly-Funded Substance Use Treatment. Rockville, MD; 2019.

62. United States Census Bureau. State Population Totals and Components of Change 2010-2019: Annual Estimates of the Resident Population for the United States [Internet]. 2019 [cited 2020 May 27]. Available from: https://www.census.gov/data/tables/time-series/demo/popest/2010s-state-total.html#par_textimage_1574439295

63. Substance Abuse and Mental Health Services Administration. National Survey of Substance Abuse Treatment Services (N-SSATS) 2015, 2016, 2017 Data on Substance Abuse Treatment Facilities. Rockville, MD; 2017.
