# Supplementary material for: Is Europe facing an opioid crisis like the United States? An analysis of opioid use and related adverse effects in 19 European countries between 2010 and 2018
Source: Eur Psychiatry. 2021 Jun 21;64(1):e47. doi: 10.1192/j.eurpsy.2021.2219 (PMC8316471; doi:10.1192/j.eurpsy.2021.2219)
Supplement: Supplementary file 1 [file epasup.zip › S0924933821022197sup003.pdf]

*Table 1 Summary of Findings*

| | High risk opioid users | Consumption of all PO's | Opioid-related hospital admissions | Opioid-related overdose mortality | Opioid treatment admissions | OST patients |
|---|---|---|---|---|---|---|
| | *(rate per 1000 population 15-64 years)* | *(year: s-DDD per 1000,000 inhabitants per day; relative % change between 2008/10 and 2016/18)* | *(year: rate per 100,000 population ≥ 15 years)* | *(year: rate per 100,000 population ≥ 15 years)* | *(year: rate per 100,000 population ≥ 15 years)* | *(year: rate per 100,000 population ≥ 15 years)* |
| Scotland | 16·2 *(total population)* | ·· | *2010: 93 2017: 118 *(total population)* | 2010: 10·9 2018: 22·7 | 2010: 124 2018: 85 | 2011: 589 2018: 555 |
| The UK | 8·4 | 2008-2010: 18376 2016-2018: 58088 216% | ·· | ·· | ·· | 2010: 301 2017: 276 |
| England | 7·2 *(total population)* | ·· | *2012: 33 2018: 35 | 2010: 3·0 2018: 4·0 *(England & Wales)* | 2010: 119 2018: 84 | ·· |
| Austria | 6·3 | 2008-2010: 16315 2016-2018: 19867 22% | 2010: 13 2017: 16 *(15-64 years)* | 2010: 4·2 2018: 3·3 *(15-64 years)* | ·· | 2010: 279 2018: 326 *(15-64 years)* |
| Ireland | 6·2 | 2008-2010: 5946 2016-2018 7061 19% | 2010: 13 2017: 18 | 2010: 4·8 2017: 7·9 | 2010: 134 2018: 104 | 2010: 258 2017: 273 |
| Italy | 6·0 | 2008-2010: 3553 2016-2018: 5083 43% | 2010: 3 2017: 1 | 2010: 0·3 2018: 0·3 | 2013: 35 2018: 31 | 2010: 200 2018: 144 |
| Latvia | 5·7 | 2008-2010: 827 2016-2018: 2102 154% | 2014: 11 2018: 11 | 2014: 0·8 2018: 0·8 | 2014: 74 2018: 81 | 2010: 13 2018: 42 |
| France | 5·0 | 2008-2010: 8827 2016-2018: 8011 -9% | *2010: 8 2018: 10 | 2010: 0·5 2017: 0·7 | 2014: 16 2018: 17 | 2010: 304 2017: 337 |
| Lithuania | 3·9 | 2008-2010: 1127 2016-2018: 1445 28% | 2010: 5 2018: 2 | 2010: 0·9 2018: 1·6 | 2013: 76 2017: 60 | 2010: 25 2018: 28 |
| Norway | 2·7 | 2008-2010: 9167 2016-2018: 11949 30% | ·· | 2010: 5·0 2018: 5·6 | ·· | 2010: 153 2017: 176 |

*Table 1 Summary of Findings*

| | | | | | | |
|---|---|---|---|---|---|---|
| Cyprus | 2·0 | 2008-2010: 704<br>2016-2018: 1630<br>132% | ·· | 2010: 1·1<br>2018: 0·8 | 2011: 56<br>2018: 52 | 2011: 9<br>2018: 20<br>*(total population)* |
| Germany | 2·0 | 2008-2010: 15314<br>2016-2018: 21167<br>38% | 2010: 6<br>2017: 4<br>*(total population)* | 2010: 0·3<br>2016: 0·3<br>*(total population)* | 2010: 40<br>2018: 8<br>*(total population)* | 2010:95<br>2018: 96<br>*(total population)* |
| The Czech Republic | 1·9 | 2008-2010: 3502<br>2016-2018: 6261<br>79% | 2010: 1<br>2018: 1 | 2010: 0·2<br>2018: 0·2 | 2010: 23<br>2018: 15 | 2010: 23<br>2018: 26 |
| The Netherlands | 1·3 | 2008-2010: 6565<br>2016-2018: 14621<br>123% | 2010: 4<br>2017: 10 | 2010: 0·3<br>2017: 0·9 | 2010: 10<br>2015: 6 | 2010: 74<br>2015: 37 |
| Estonia | ·· | 2008-2010: 706<br>2016-2018: 969<br>37% | ·· | 2010: 8·0<br>2015: 6·6 | ·· | 2010: 89<br>2018: 90 |
| Northern Ireland | ·· | ·· | *2010: 53<br>2018: 78 | 2010: 3·6<br>2017: 5·8 | 2010: 29<br>2016: 67 | 2010: 45<br>2017: 66 |
| Switzerland | ·· | 2008-2010: 11140<br>2016-2018: 16134<br>45% | *2012: 32<br>2018: 23 | ·· | 2010: 22<br>2018: 31 | 2010: 227<br>2018: 196 |
| Slovakia | ·· | 2008-2010: 2972<br>2016-2018: 5528<br>86% | *2010: 7<br>2918: 4 | 2010: 0·2<br>2018: 0·2 | 2014: 54<br>2018: 18 | 2010: 13<br>2017: 13 |
| Russia | ·· | 2008-2010: 85<br>2016-2018: 237<br>179% | ·· | ·· | 2010: 61<br>2017: 18<br>*(total population)* | ·· |
| Belgium | ·· | 2008-2010: 22769<br>2016-2018: 20758<br>-9% | 2010: 6<br>2018: 9 | 2010: 0·3<br>2016: 0·2 | 2015: 28<br>2018: 22 | 2010: 204<br>2017: 183<br>(≥ *18 years)* |
| The US | ·· | 2008-2010: 18376<br>2016-2018: 17853<br>-6% | *2010: 197<br>2017: 300<br>*(total population)* | 2010: 6·8<br>2018: 14·6<br>*(standard population)* | 2010: 143<br>2017: 210<br>*(total population)* | 2011:393<br>2017: 417<br>*(total population)* |

"··" = no data available

Opioid related hospital admissions: * = same definition used to define data

If a different population sample (not 15 years and older) was used to calculate the rates this is given in brackets following the data from that country.
